# HDAC7 induction combined with standard-of-care chemotherapy provides a therapeutic advantage in t(4;11) infant B-cell acute lymphoblastic leukemia

**DOI:** 10.1186/s40364-025-00810-1

**Published:** 2025-07-28

**Authors:** Oriol de Barrios, Ingrid Ocón-Gabarró, Mar Gusi-Vives, Olga Collazo, Ainara Meler, Paola A. Romecín, Alba Martínez-Moreno, Juan Ramón Tejedor, Mario F. Fraga, Pauline Schneider, Michela Bardini, Giovanni Cazzaniga, Rolf Marschalek, Ronald W. Stam, Clara Bueno, Pablo Menéndez, Maribel Parra

**Affiliations:** 1 Lymphocyte Development and Disease Group, Josep Carreras Leukaemia Research Institute (IJC), Ctra de Can Ruti, Camí de les Escoles, s/n, 08916 Badalona, Barcelona, Spain; 2 Doctoral Program in Biomedicine, Universitat de Barcelona (UB), Barcelona, Spain; 3 Center for Immuno-Oncology, Center for Cancer Research, National Cancer Institute, Bethesda, MD USA; 4 Josep Carreras Leukaemia Research Institute, School of Medicine, University of Barcelona, 08036 Barcelona, Spain; 5 Nanomaterials and Nanotechnology Research Center (CINN-CSIC), Institute of Oncology of Asturias (IUOPA), Health Research Institute of Asturias (ISPA), 33012 Oviedo, Spain; 6 CIBER-ER-ISCIII, Madrid, Spain; 7 Princess Maxima Center for Paediatric Oncology, Utrecht, The Netherlands; 8 Tettamanti Center, Fondazione IRCCS San Gerardo Dei Tintori, Monza, Italy; 9 School of Medicine and Surgery, University of Milano-Bicocca, Milan, Italy; 10 Institute of Pharmaceutical Biology/DCAL, Goethe-University, Frankfurt, Germany; 11 Red Española de Terapias Avanzadas (TERAV), Instituto de Salud Carlos III (ISCIII), Madrid, Spain; 12 Department of Biomedicine, School of Medicine, University of Barcelona, Barcelona, Spain; 13 Institució Catalana de Recerca i Estudis Avançats (ICREA), Barcelona, Spain; 14 Centro de Investigación Biomédica en Red (CIBERONC), ISCIII, Madrid, Spain

**Keywords:** Infant t(4;11) B-ALL, HDAC7, Combinatorial therapy, Menin-1 inhibitors

## Abstract

**Background:**

Infants diagnosed with B cell acute lymphoblastic leukemia (B-ALL) and t(4;11) chromosomal rearrangement display poor therapeutic response, associated to the low expression of B lymphocyte factor HDAC7. This study was conceived to identify a therapeutic strategy for t(4;11) B-ALL that restores optimal HDAC7 expression.

**Methods:**

A multiomics approach in a large infant pro-B-ALL cohort was employed to identify HDAC7’s repression mechanism. These data, combined with cell culture assays in a variety of pro-B-ALL cell lines with differential HDAC7 levels, led us to define a novel combination therapy. Murine leukemia models and ex vivo assays using patient-derived xenografts (PDX) were employed to assess the benefits of this therapy when incorporated to glucocorticoid-based chemotherapy.

**Results:**

Our data demonstrates that HDAC7 is epigenetically silenced by EZH2 and KMT2A::AFF1 fusion protein. Remarkably, the Menin-1 inhibitor MI-538 restores HDAC7 expression, and the effect is enhanced by class I HDAC inhibitor chidamide. This treatment drives leukemic pro-B cells towards a more differentiated state and impairs aberrant proliferation in an HDAC7-dependent manner. This newly identified therapy increases glucocorticoid sensitivity of PDX cells ex vivo, by repressing RUNX2 transcription factor. Finally, combining MI-538 and chidamide with standard chemotherapy reduces PDX cells engraftment in vivo and delays relapse.

**Conclusions:**

The combined therapy proposed, based on Menin-1 inhibition, improves t(4;11) B-ALL cells’ response to standard therapy, an effect partially mediated by HDAC7 induction. Therefore, this novel therapy opens a new field for personalized treatments in high-risk leukemia, especially for infants presenting low expression of HDAC7 B cell factor.

**Supplementary Information:**

The online version contains supplementary material available at 10.1186/s40364-025-00810-1.

## Background

Infant acute lymphoblastic leukemia (ALL) frequently originates at the B-cell progenitor (pro-B cell) stage [1, 2]. The involvement of *KMT2A* in chromosomal rearrangements is often associated with poor response to conventional treatment and increased relapse rate, leading to poor outcomes [2–4]. Current therapy consists of an initial induction phase with prednisone, vincristine and L-asparaginase, followed by a consolidation phase upon remission [5, 6]. The addition of blinatumomab to standard chemotherapy has recently been shown to improve the outcome of *KMT2A*-rearranged infant B-ALL [7]. However, infants with the t(4;11) translocation between *KMT2A* and *AFF1*, giving rise to KMT2A::AFF1 fusion (classically known as MLL-AF4), have a higher associated risk [2, 4] and, importantly, represent a vast majority of pro-B-ALL patients diagnosed at ≤ 1 year of age [4, 8].

KMT2A is an important chromatin modifier that is involved in differentiation processes. The KMT2A protein complex is recruited to target gene promoters via interaction of the N-terminal triade of KMT2A/Menin-1/LEDGF. These promoter regions are epigenetically modified through C-terminal SET domain complex by H3K4me3 histone signature. This is of particular interest for t(4;11) translocation, where the resulting KMT2A::AFF1 fusion protein strongly overexpresses a set of target genes [9–11]. Other chromatin remodelers such as class I histone deacetylases (HDACs) are recruited to the KMT2A::AFF1 fusion protein regulating chromatin accessibility [12, 13]. Importantly, Menin-1 binding to KMT2A is required for oncogenic activity, whereas its downregulation provokes the loss of leukemic transformation [14].

Despite the challenges in regulating the KMT2A::AFF1 activity, recent advancements have demonstrated the potential of blocking its essential cofactors [15]. Among KMT2A partners, Menin-1 has emerged as a druggable candidate, followed by the development of small molecules that block its direct interaction with the N-terminal domain of KMT2A [14, 15]. The chemical structure of Menin-1 inhibitors (MI) has evolved, and more effective molecules are currently in clinical trials for the treatment of acute myeloid leukemia (AML) [16].

Infant leukemia arises at embryonic stages and is mediated by alterations of defined genetic programs that steer the differentiation of B cells [2, 17]. In this context, the role of key transcription factors that promote B cell development are well established [18–20]. However, altered expression and/or mutations that disrupt the delicate balance between these factors are associated with the manifestation of hematopoietic malignancies [21, 22]. A clear example of a B cell factor whose dysregulation results in the leukemogenic behavior of infant pro-B-ALL cells is the class IIa histone deacetylase HDAC7, a transcriptional modulator that represses lineage inappropriate genes, such as myeloid genes, at the pro-B stage [23, 24]. Consequently, HDAC7 expression is necessary for pro-B cells identity and their transition to more differentiated pre-B lymphocytes [23]. Conversely, dysregulation or underexpression of HDAC7 is associated with higher proliferation of B cell precursors [25, 26], and was recently associated to poorer prognosis and higher treatment resistance in infant t(4;11) pro-B-ALL [26].

Here, we demonstrate that low expression of HDAC7 in t(4;11) pro-B-ALL cells is mediated by epigenetic mechanisms involving KMT2A::AFF1 and chromatin remodeler EZH2. These findings led us to develop a promising combinatorial treatment that restores HDAC7 transcription. This novel strategy induces B cell differentiation and lineage commitment, conferring a less malignant phenotype. The newly proposed therapy also increases the sensitivity of primary leukemic cells to glucocorticoids and shows promising results in vivo when combined with standard chemotherapy.

## Methods

### Use of human pro-B-ALL primary samples in animal procedures

Patient samples from KMT2A germline and t(4;11) pro-B-ALL infants are cryopreserved and legally stored in INTERFANT06 protocol repository biobanks, with the authorization from patients’ legal representatives, in accordance with the Declaration of Helsinki. The experimental work proposed in this study involving primary human samples has been approved by the IRB board at Germans Trias i Pujol hospital, at Can Ruti Campus in Badalona (approval code: PI21/01451). All animal procedures involved are approved by the Animal Research Ethics Committee of CMCiB-IGTP facility (20–004-JRU-P5) and the Animal Research Commission from the Government of Catalonia (CEA/10473_MR2/P5).

### Human cell lines

Human SEM-K2, RS4;11 and REH cell lines were kindly provided by Dr. Manel Esteller (Josep Carreras Leukaemia Research Institute, Badalona, Spain) and originally acquired from DSMZ (Leibniz Insittute, Braunschweig, Germany). ALL-PO cells were kindly provided by Dr. Giovanni Cazzaniga (University of Milano-Bicocca, Monza, Italy). All cell lines were authenticated by STR DNA fingerprinting at Genomics Unit of Germans Trias i Pujol Research Institute (IGTP, Badalona, Spain), using the AmpFLSTR Identifiler Plus PCR amplification kit from ThermoFisher Scientific (Waltham, MA, USA).

### Statistical analysis

Statistical comparisons between experimental conditions and the potential additive effects of drug combinations used were analyzed by using GraphPad Prism (version 8.3 GraphPad Software, La Jolla, CA, USA) and presented as means ± SEM. Non-parametric Mann–Whitney test was used in data analysis, except for the analysis of normalized RNA-seq counts data, where Student’s t-test was employed after assessing normal distribution of data with Shapiro–Wilk test. A *p* value < 0.05 was considered statistically significant. ns: *p* > 0.05, *: *p* < 0.05, **: *p* < 0.01, ***: *p* < 0.001. The number of mice or replicates used in every experiment are included in the respective figure legend.

The rest of Material and Methods data, as well as Supplementary Tables S1-S3, are available at *Supplementary Information* document.

## Results

### Forced restoration of HDAC7 reduces the aggressiveness of t(4;11) B-ALL cells and improves survival in vivo

**Fig. 1:**
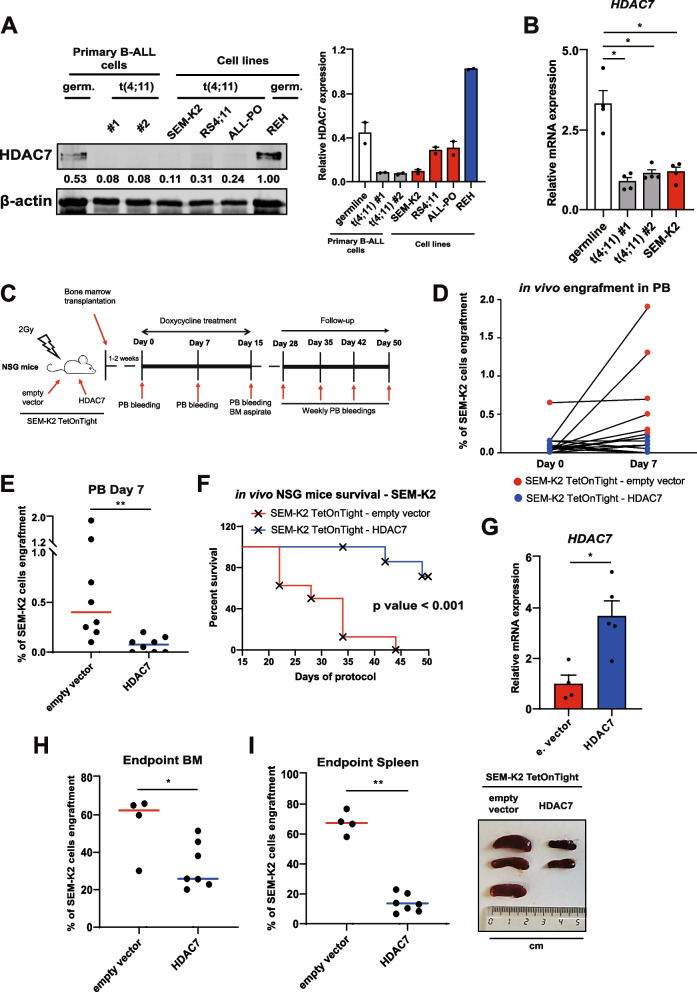
HDAC7 reduces leukemogenic capacity of t(4;11) pro-B-ALL cells and improves survival in vivo. **A** Protein levels (*left panel*) and associated quantification (*right panel*) of HDAC7 in PDX cells obtained from one germline (abbreviated as germ.) *KMT2A* and two t(4;11) pro-B-ALL (patients #1 and #2), compared to that in cell lines SEM-K2, RS4;11, ALL-PO and REH. Cells from patient #1 were obtained at diagnosis stage, while patient #2 cells were obtained after relapse. Patient #1 corresponds to ALL-KA1_26 in Supplementary Figs. S1A and S1B, whereas patient #2 and *KMT2A* germline patient are not included in this dataset. β-actin was used as loading control. Data in *right panel* corresponds to the average quantification of two independent blots. **B** Expression of human *HDAC7* mRNA by qRT-PCR in PDX from *KMT2A* germline and t(4;11) pro-B-ALL samples used in (A), and compared to SEM-K2 cell line (*n* = 4 per condition). Human *GAPDH* and *RPL38* were used as housekeeping genes. **C** Experimental design of in vivo leukemogenesis assays performed in immunodeficient NSG mice. **D** Evolution of PB engraftment for empty vector- (red) and HDAC7-injected (blue) mice between Day 0 and Day 7. **E** Average engraftment of SEM-K2 TetOnTight-HDAC7 (and corresponding empty vector cells) in peripheral blood (PB) 7 days after treatment initiation (*n* = 8 mice per group). **F** Overall survival of mice injected with empty vector and HDAC7 SEM-K2 TetOnTight cells after finishing doxycycline treatment. Time points only show protocol days after completing doxycycline administration (*n* = 8 mice per group). **G** Expression of human *HDAC7* mRNA by qRT-PCR in bone marrow after mice euthanasia at experimental endpoint (*n* = 4 mice, empty vector group; *n* = 5 mice, HDAC7 group). Human *GAPDH* and *RPL38* were used as housekeeping genes. **H** Median engraftment of SEM-K2 TetOnTight-empty vector (red, *n* = 4 mice) and HDAC7 (blue, *n* = 7 mice) in BM at time of euthanasia for each mouse, or at experimental endpoint (Day 50). **I** As in (**H**), but for spleen engraftment (*left panel*). *Right panel* shows a representative picture of spleens (measured in cm) at Day 35 after treatment initiation. Mice transplanted with empty vector cells were humanely euthanized, whereas mice transplanted with HDAC7 cells were euthanized for comparison. Results in (**A-B**) and (**G**) show average ± SE. Statistical significance is indicated as: *p < 0.05; **p < 0.01; ***p < 0.001; or n.s. non-significant

HDAC7 represents a novel opportunity for targeted therapies in infant pro-B-ALL, since its expression levels in *KMT2A::AFF1*-rearranged patients correlate with an improved prognosis [26]. Indeed, the transcriptome of blasts from infants with t(4;11) pro-B-ALL clustered away from peers with a germline (i.e. unaltered) *KMT2A* gene [27] (Supplementary Fig. S1A). Infants presenting KMT2A::AFF1 alteration were split by median *HDAC7* expression and allocated in *HDAC7*_high_ and *HDAC7*_low_ groups. Despite both groups display significantly lower *HDAC7* levels than infants lacking *KMT2A* alterations [26], *HDAC7*_high_ t(4;11) leukemia cases grouped significantly with germline *KMT2A* as compared with HDAC7_low_ patients [OR = 10.286, 95% CI (1.018–103.953), *p* < 0.05] (Supplementary Fig. S1B). Accordingly, HDAC7 showed differential expression at the protein and mRNA levels in both cell lines and primary leukemic cells when comparing t(4;11) pro-B-ALL samples with germline *KMT2A* cells (Fig. 1A, B and Supplementary Fig. S1C). Low HDAC7 expression in t(4;11) primary cells is comparable with that observed in SEM-K2, RS4:11 and ALL-PO t(4;11) pro-B-ALL cell lines. Primary cells harboring germline *KMT2A* showed higher HDAC7 expression, as well as germline REH cell line (Fig. 1A, B and Supplementary Fig. S1C), correlating with the improved survival observed in patients with unaltered *KMT2A* [6].

As the basal expression of HDAC7 was comparable between primary t(4;11) pro-B-ALL cells and cell lines, we used the SEM-K2 cells in an in vivo model to determine whether restoring HDAC7 mitigates leukemogenic potential and improves survival. We employed previously generated stable SEM-K2 TetOnTight-HDAC7 cells with doxycycline-inducible HDAC7 expression (Supplementary Fig. S1D), which present an impaired proliferation and increased apoptosis in vitro [26]. After intra bone marrow (BM) transplantation of these stably modified cells or SEM-K2 TetOnTight-empty vector counterparts in NSG immunodeficient mice, doxycycline was administered in drinking water for two weeks (Fig. 1C). Peripheral blood (PB) analysis on day 7 post-treatment showed that mice injected with SEM-K2 TetOnTight-HDAC7 cells had reduced engraftment (Fig. 1D, E and Supplementary Fig. S1E). Mice bearing HDAC7-overexpressing cells still presented reduced PB engraftment at day 35, three weeks after treatment completion (Supplementary Fig. S1F), which remarkably translated into a substantially improved overall survival (Fig. 1F). At the experimental endpoint, mRNA obtained from the BM confirmed the induction of *HDAC7* after doxycycline administration in mice transplanted with SEM-K2 TetOnTight-HDAC7 cells (Fig. 1G), correlating with the lower presence of human leukemic cells both in BM and spleen (Fig. 1H, I). These findings corroborate the beneficious effects of restoring HDAC7 expression in t(4;11) pro-B-ALL cells. In consequence, we aimed to study the molecular mechanisms underlying HDAC7 inhibition in this malignancy and to investigate how to precisely restore its physiological levels, avoiding the use of forced exogenous overexpression.

### HDAC7 is epigenetically silenced in infant t(4;11) B-ALL

**Fig. 2:**
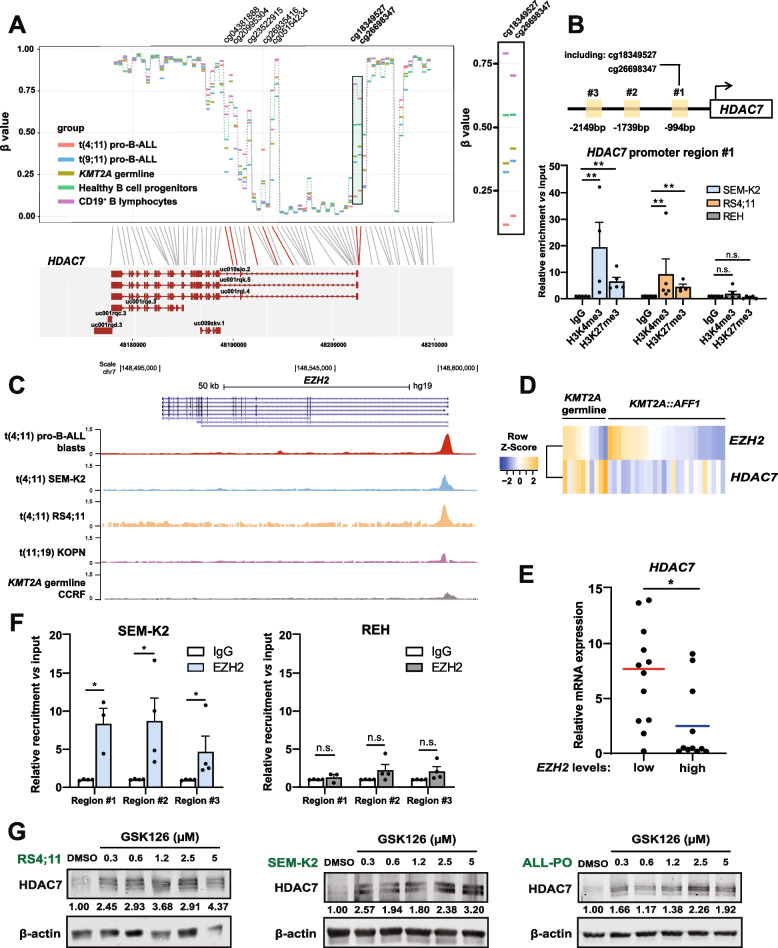
Chromatin remodeler EZH2 represses HDAC7 in t(4;11) pro-B-ALL cells. **A** Relative quantification of the methylation levels of the CpG sites within human *HDAC7* gene and its corresponding promoter region in pro-B-ALL cells with germline *KMT2A* (*n* = 20), and t(4;11) and t(9;11) translocations (*n* = 37 and *n* = 12, respectively). Data from healthy B cell progenitors (*n* = 6) and CD19^+^ B lymphocytes (*n* = 1) is also included. CpG sites where t(4;11) pro-B-ALL patients present differential methylation are labeled at the top of the chart. Panel on the right zooms the green-shadowed region located in *HDAC7* promoter containing two differentially-methylated CpG sites (cg18349527 and cg26698347). Data is publicly available in ArrayExpress (accession number: E-MTAB-8505). **B** In *top panel*, schematic view of human *HDAC7* promoter, showing three different regions (#1, #2 and #3) analyzed by ChIP-qPCR in infant B-ALL cells. Region #1 includes differentially methylated CpG sites highlighted in (**A**). *Bottom panel*, ChIP-qPCR data for region #1 of *HDAC7* promoter after immunoprecipitation with H3K4me3 and H3K27me3 antibodies in SEM-K2, RS4;11 and REH cells, along with corresponding anti-rabbit IgG, as control (*n* = 4–5 experiments per cell line). **C** ChIP sequencing data for *EZH2* gene in t(4;11) pro-B-ALL primary blasts (in red) and the following cell lines: SEM-K2 (blue), RS4;11 (orange), t(11;19) KOPN (purple) and *KMT2A* germline CCRF (grey). Binding peaks are shown for each track after immunoprecipitation with KMT2A antibodies. Results obtained from publicly available data (GSE202449, GSE74812, GSE83671 and GSE38403). **D** Heatmap for mRNA counts of *EZH2* and *HDAC7* in *KMT2A* germline and t(4;11) pro-B-ALL patients from Supplementary Figs. S1A and S1B (*n* = 10 and 26, respectively). **E** Average mRNA expression of *HDAC7* in t(4;11) pro-B-ALL (*n* = 22), after dividing patients by median expression of *EZH2* in *EZH2*_high_ and *EZH2*_low_ groups (*n* = 11 per group). *GAPDH* and *RPL38* were used as housekeeping genes. **F** ChIP-qPCR data for the three regions of *HDAC7* promoter shown in (**B**), in SEM-K2 and REH cells (*n* = 3–4 experiments per cell line), after immunoprecipitation with EZH2 antibody, and the corresponding IgG as control. **G** Protein levels of HDAC7 in RS4;11, SEM-K2 and ALL-PO cell lines, at increasing doses of GSK126, or DMSO solvent as control. β-actin was used as loading control. Results in (**B**) and (**F**) are shown as average ± SE. Statistical significance is indicated as: *p < 0.05; **p < 0.01; ***p < 0.001; or n.s. non-significant

To elucidate the potential mechanism(s) responsible for the lower expression of HDAC7 in blasts from infants with t(4;11) pro-B-ALL, we first analyzed the methylation status of CpG islands throughout the HDAC7 regulatory regions and gene body. Unexpectedly, a specific promoter region within the vicinity of the transcriptional start site was clearly hypomethylated in t(4;11) pro-B-ALL cases when compared with other pro-B-ALL genotypes (Fig. 2A and Supplementary Fig. S2A).

This differential pattern of DNA methylation may not be sufficient to fully explain the inhibited expression of HDAC7 in t(4;11) pro-B-ALL, as histone modifications may also exert a complementary function [28]. Thus, we analyzed the promoter region of *HDAC7* (#1, located at −994 bp from the transcription start site) containing the two CpG islands that were differentially hypomethylated (cg18349527 and cg26698347 in Fig. 2A), along with two additional regions in the vicinity (regions #2 and #3, located at −1739 bp and −2149 bp, respectively), for the presence of both activating and repressive histone marks in SEM-K2 and RS4;11, and the *KMT2A* germline REH cells. H3K4me3, histone mark associated to promoter activation [13], was significantly enriched at the *HDAC7* promoter only in t(4;11) pro-B-ALL cells. We also analyzed the methylation status of the H3K27 residue, frequently altered in cancer [29]. This repressive mark was found to be enriched specifically at the *HDAC7* promoter in t(4;11) pro-B-ALL cells but not in germline cells (Fig. 2B and Supplementary Fig. S2B), suggesting a possible mechanism for epigenetically-mediated HDAC7 repression. REH cells showed a much higher enrichment of the H3K27ac mark at the *HDAC7* promoter (Supplementary Fig. S2C), correlating with the higher expression of HDAC7 in germline *KMT2A* cells (Fig. 1A, B).

Overall, a global increase in H3K27me3 levels and an additional histone repressive mark (H3K9me3) was observed in SEM-K2 and RS4;11 cell lines but not in REH cells, which were enriched for H3K27ac, associated with transcriptionally active chromatin (Supplementary Fig. S2D). Regarding H3K4me3 activation histone mark, a slight enrichment was found in SEM-K2 and RS4;11 cells, indicating higher KMT2A activity (Supplementary Fig. S2E). Collectively, these data suggest that HDAC7 is epigenetically silenced through a bivalent chromatin signature in high-risk infant t(4;11) cells, regulated by histone methylation/acetylation balance.

### HDAC7 silencing in infant t(4;11) pro-B-ALL is mediated by EZH2 chromatin remodeler

The polycomb repressive complex 2 (PRC2) member EZH2 is the main enzyme for methylating H3K27 residues in cancer, leading to gene silencing [30, 31]. Therefore, the enrichment of H3K27me3 at the *HDAC7* promoter prompted us to investigate whether EZH2 may be responsible for its silencing in t(4;11) pro-B-ALL cells. We first analyzed a possible correlation between EZH2 and HDAC7 expression. We classified the RNA-seq dataset described earlier (Supplementary Figs. S1A and S1B) based on *EZH2* expression levels (*EZH2*_low_ and *EZH2*_high_)_,_ and found that t(4;11) pro-B-ALL patients classified as *EZH2*_high_ tended to cluster separately from germline *KMT2A* patients [OR = 0.291, 95% CI (0.045–1.898), *p* = 0.197] (Supplementary Fig. S2F), suggesting that high levels of *EZH2* shift the transcriptional profile of infants with t(4;11) pro-B-ALL away from germline *KMT2A* individuals, oppositely to the effect caused by an elevated *HDAC7* expression.

We next investigated whether aberrant t(4;11)-derived KMT2A::AFF1 protein is involved in the direct regulation of *EZH2*. Analysis of publicly available ChIP-seq data [11, 32–34] showed that KMT2A is bound to *EZH2* regulatory promoter region in t(4;11) cells reinforcing the hypothesis of direct *EZH2* activation by the KMT2A::AFF1 protein (Fig. 2C). Moreover, direct targeting of *KMT2A::AFF1* expression drastically reduced binding to EZH2 promoter region (Supplementary Fig. S2G). Notably, RNA-seq data revealed that high presence of *EZH2* transcript within t(4;11) pro-B-ALL patients corresponds to the subgroup of cases with the lowest *HDAC7* expression (Fig. 2D). We validated these results by qRT-PCR, finding a significant inverse correlation between the expression of both genes after dividing patients into two groups based on *EZH2* median expression (Fig. 2E).

Next, we examined the expression of potential EZH2 targets [35, 36] in patients with germline *KMT2A* and t(4;11) pro-B-ALL. We found EZH2-repressed genes substantially downregulated in t(4;11) patients, while EZH2-activated targets were contrarily induced (Supplementary Figs. S2H and S2I). Moreover, EZH2 proved to be recruited to *HDAC7* promoter in SEM-K2 cells, but not in *KMT2A* germline REH cells (Fig. 2F). This confirms that HDAC7 may be directly repressed by EZH2 in infant t(4;11) pro-B-ALL and suggests EZH2 blockade as a promising therapeutic strategy.

### Blockade of EZH2 activity triggers HDAC7 expression

Novel drugs that block EZH2 activity have been developed and are currently in clinical trials [30, 37]. Aiming to determine whether EZH2 inhibition could restore HDAC7 expression, we selected two EZH2 inhibitors (GSK126 and EPZ6438) and exposed t(4;11) pro-B-ALL cells to increasing doses of these drugs. The specificity of these compounds was confirmed by the reduction of global H3K27me3 levels in t(4;11) pro-B-ALL cells. These assays revealed reduced levels of H3K27me3 even at the lowest doses used. Interestingly, this global decrease in H3K27 methylation induced EZH2 levels, probably due to compensatory mechanisms (Supplementary Fig. S3A). Analysis of protein expression after treatment of RS4;11, SEM-K2 and ALL-PO cells with both drugs revealed an induction of HDAC7 expression, even at doses below 1 µM (Fig. 2G and Supplementary Fig. S3B). As a confirmation of the compounds effect, EZH2 targets were activated after treatment of SEM-K2 cells, specifically *PRDM1* and *CCND1* [30] (Supplementary Figs. S3C and S3D).

Suprisingly, GSK126 or EPZ6438 treatment did not reduce cell viability of SEM-K2 cells and, in the case of RS4;11, only very high doses of GSK126 significantly impaired cell viability (Supplementary Fig. S4A). These unexpected results led us to investigate whether the blockade of EZH2 may lead to the activation of alternative oncogenic pathways, such as FLT3 and its downstream signaling, previously reported for childhood leukemia [38]. In t(4;11) pro-B-ALL context, the exposure of SEM-K2 cells to GSK126 and EPZ6438 resulted in increased expression of FLT3 (Supplementary Fig. S4B) and its downstream signaling, mediated by STAT5 phosphorylation (Supplementary Figs. S4C and S4D). This unwanted non-specific effect led us to discard EZH2 inhibition as an optimal strategy for HDAC7 activation.

### Combined inhibition of KMT2A::AFF1 cofactors reduces leukemogenesis of t(4;11) pro-B-ALL cells

**Fig. 3:**
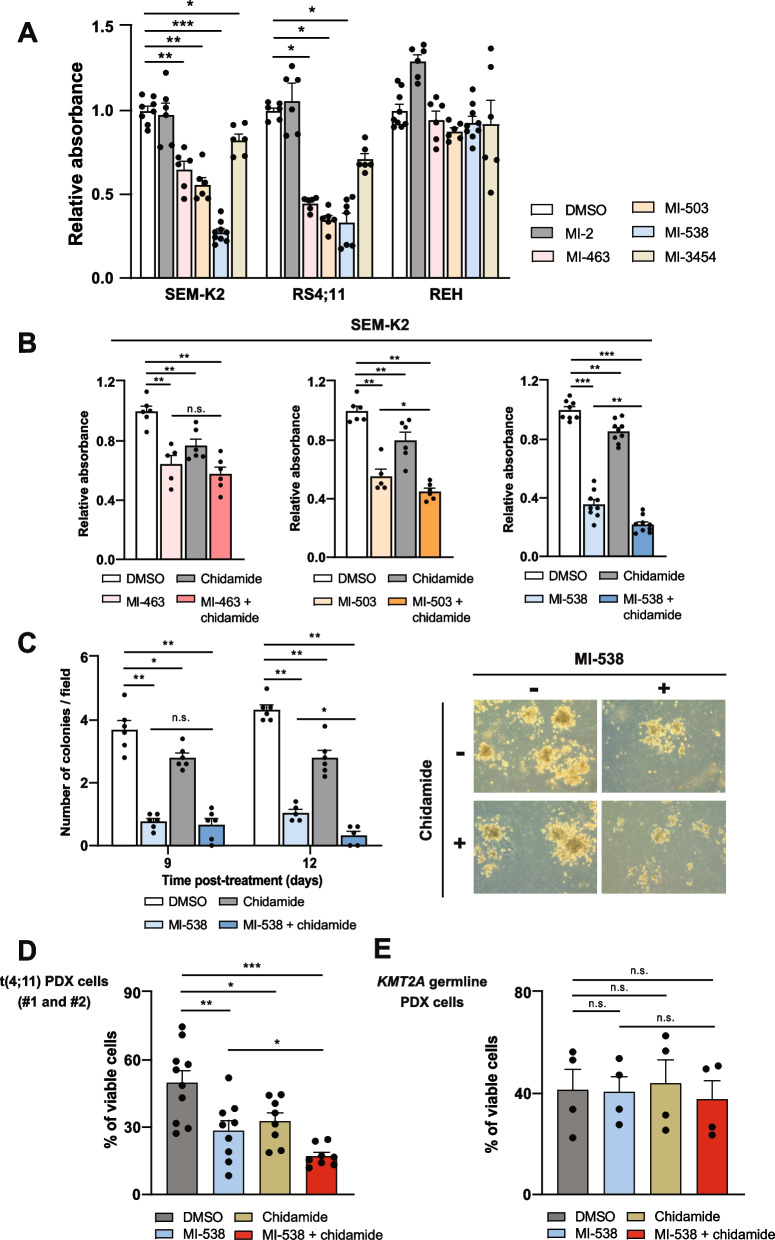
Inhibition of KMT2A::AFF1 cofactors reduces proliferation and leukemogenic capacity of t(4;11) pro-B-ALL cells. **A** Cell viability by MTT assays in SEM-K2, RS4;11 and REH cells, treated for 6 days with 1 µM of Menin-1 inhibitors MI-2, MI-463, MI-503, MI-538 and MI-3454, or DMSO solvent as control (*n* = 6–9 per condition and cell line). **B** Cell viability by MTT assays in SEM-K2 cells. Where indicated, cells were treated with Menin-1 inhibitors MI-463, MI-503 or MI-538 (1 µM, 6 days) and/or chidamide (1 µM, 48 h). Results are presented as relative absorbance for the differences between values measured at 560 and 750 nm (*n* = 9 for MI-538 and *n* = 6 for MI-463 and MI-503). **C** Quantification of colony formation capacity of SEM-K2 cells in Methocult® media at different time points (days 9 and 12) (*left panel*). Cells were seeded at 1,000 cells/plate concentration and treated with MI-538 and/or chidamide (1 µM each) for up to 14 days. Experiment was repeated four times (with each condition in triplicate) and the quantification represents the average of five different microscopic fields per plate (*n* = 6 independent experiments per condition). *Right panel*, representative pictures of colonies in each experimental condition. Magnification 100X. **D** Cell viability assay for PDX pro-B-ALL cells obtained from t(4;11) samples #1 and #2 previously shown in Fig. 1A-B, after 36-48 h in culture. Results are displayed as percentage of Annexin V/7-AAD double negative cells and each point represents a PDX sample (*n* = 8–10 samples per condition). Where indicated, cells were treated with MI-538 1 µM, chidamide 1 µM or the combination of both drugs. **E** As in (**D**), but for PDX cells obtained from *KMT2A* germline pro-B-ALL patient (*n* = 4 samples per condition). Results in panels (**A**-**E**) are shown as average ± SE. Statistical significance is indicated as: * or ^#^ *p* < 0.05; ** or ^##^ *p* < 0.01; ***p < 0.001; or n.s. non-significant

The lack of success in reducing leukemogenic capacity of t(4;11) pro-B-ALL cells with EZH2 inhibitors, led us to speculate on directly interfering with the KMT2A::AFF1 activity. We first tested a panel of inhibitors of the KMT2A::AFF1 cofactor Menin-1 (MI), covering a range of chemical structures, with the aim of defining the compound with stronger activity in t(4;11) pro-B-ALL cells. Particularly, MI-538 had the strongest activity in reducing the viability of pro-B-ALL cells. Notably, the activity of these compounds was restricted to t(4;11) pro-B-ALL cells, as germline REH cells (HDAC7_high_) failed to respond to any tested MI (Fig. 3A). Determination of the IC50 concentration for MI-538 after 72 h of treatment revealed significant differences between t(4;11) and germline *KMT2A* pro-B-ALL cells (Supplementary Fig. S5A).

In addition to Menin-1 inhibition, class I HDACs are also fundamental to the transcriptional activity driven by KMT2A::AFF1 [39]. The ability to specifically repress class I HDACs can trigger HDAC7 by preventing its repression mediated by HDAC1 and HDAC2, but also by impairing its binding to HDAC3 [25, 39]. Considering the potential benefits of a combination therapy, we included the class I HDAC inhibitor chidamide, with potent activity to simultaneously repress HDAC2 and HDAC3 expression in SEM-K2 cells (Supplementary Fig. S5B). Chidamide showed a weak effect when used as a single agent. However, its combination with MI, especially with MI-538, provided an additive effect (Fig. 3B and Supplementary Fig. S5C). Notably, this combination showed very little or no ability to reduce the viability of germline *KMT2A* REH cells (Supplementary Fig. S5D) and was therefore selected for further study. Additionally, colony formation of SEM-K2 cells was substantially reduced in cells treated with MI-538 alone or in combination with chidamide (Fig. 3C and Supplementary Fig. S5E).

The additive effect of chidamide and MI-538 was confirmed using t(4;11) patient-derived xenograft (PDX) cells which showed, in contrast to *KMT2A* germline PDX cells, a significantly reduced viability ex vivo when treated with MI-538 plus chidamide (Fig. 3D, E). According to HDAC7 levels shown in Fig. 1A, B, these results demonstrate that combining MI-538 with chidamide is selectively effective in those cases with lower HDAC7, associated to a poorer outcome.

### Reduced viability of t(4;11) B-ALL cells under MI-538/chidamide combination is mediated by EZH2-HDAC7 axis

**Fig. 4:**
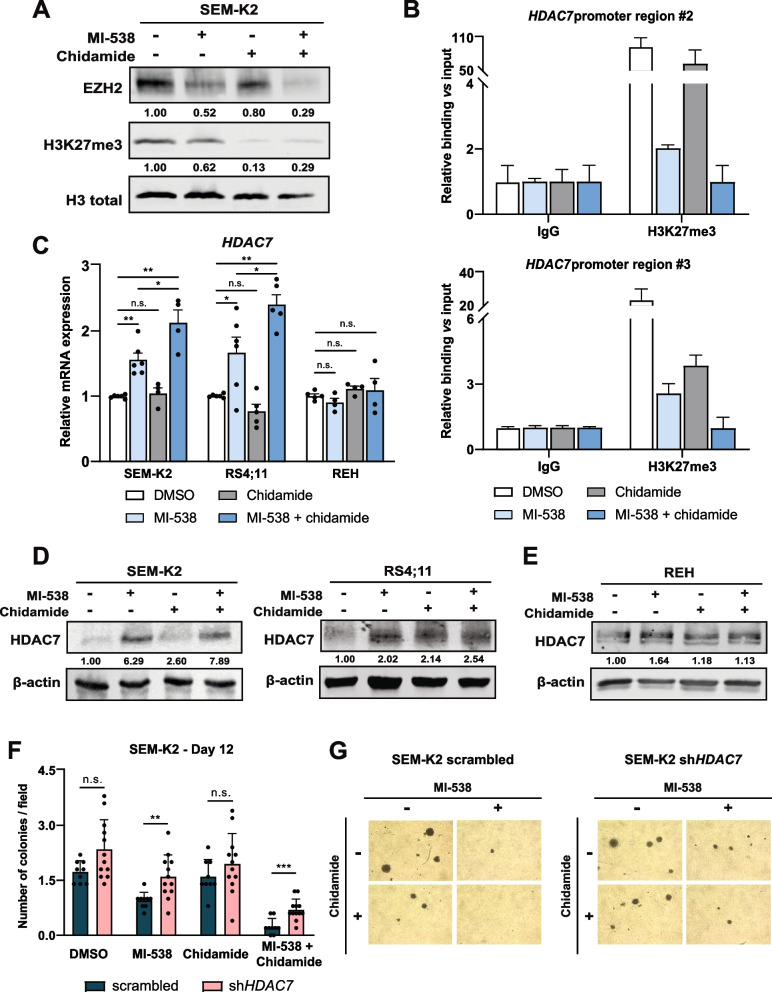
Combinatorial MI-538 + chidamide treatment impairs t(4;11) pro-B-ALL cells viability by reverting EZH2-mediated HDAC7 repression. **A** Protein levels of EZH2 and total H3K27me3 in SEM-K2 cells treated with MI-538 (1 µM, 6 days) and/or chidamide (1 µM, 48 h), or DMSO solvent as control. Total H3 protein was used as loading control. **B** ChIP-qPCR data for regions #2 (*top panel*) and #3 (*bottom panel*) of *HDAC7* promoter (as shown in Fig. 2B) after immunoprecipitation with H3K27me3 antibody in SEM-K2 cells treated with MI-538 and chidamide (or DMSO), along with corresponding anti-rabbit IgG, as control. Data shown is representative of 3 independent ChIP-qPCR experiments. **C** Expression of *HDAC7* mRNA by qRT-PCR in SEM-K2, RS4;11 and REH cells treated with 1 µM of MI-538 for 6 days and/or 1 µM of chidamide for 48 h, compared to DMSO as solvent control. *GAPDH* and *RPL38* were used as housekeeping genes (*n* = 4–6 per condition and cell line). **D** Protein levels of HDAC7 in SEM-K2 (*left panel*) and RS4;11 (*right panel*) cells treated with MI-538 (1 µM, 6 days) and/or chidamide (1 µM, 48 h), or DMSO solvent as control. β-actin was used as loading control. **E** As in (**D**), but with REH cells. **F** Quantification of colony formation capacity of SEM-K2-scrambled and SEM-K2-sh*HDAC7* cells in Methocult® media after 12 days in culture. Cells were seeded at 1,000 cells/plate concentration and treated with MI-538 and/or chidamide (1 µM each) for up to 14 days. Experiment was repeated four times (with each condition in triplicate) and the quantification represents the average of five different microscopic fields per plate (*n* = 4 experiments per condition, values of experimental triplicates are shown). **G** Representative pictures of colonies in (**F**) for each experimental condition. Magnification 40X. Results in (**B**), (**C**) and (**F**) are shown as average ± SE. Statistical significance is indicated as: *p < 0.05; **p < 0.01; ***p < 0.001; or n.s. non-significant

Having established the MI-538 and chidamide combined therapy, we evaluated whether it also affected EZH2 levels in SEM-K2 and REH cells. As expected from the binding of KMT2A::AFF1 to EZH2 promoter (Fig. 2C), the combinatorial therapy greatly reduced *EZH2* expression in SEM-K2 and RS4;11 cells, while remained unchanged in REH cells (Fig. 4A and Supplementary Fig. S6A). The changes in EZH2 expression resulted in a substantial reduction of its methyltransferase activity, shown by a global reduction of H3K27me3 marks. In fact, single treatment with chidamide also abolished H3K27me3 (Fig. 4A). The reduction in H3K27me3 levels, which mirrors the inhibition of EZH2 expression, was also reflected in regions #2 and #3 of the *HDAC7* promoter (Fig. 4B), indicating that a combined MI-538 + chidamide treatment could reverse the repressive chromatin conformation of this region.

Next, we assessed *HDAC7* mRNA levels in SEM-K2 and RS4;11 cells after combined treatment. MI-538 induced the expression of *HDAC7* and this was further enhanced by the addition of chidamide in both cell lines (Fig. 4C), demonstrating an additive effect. By contrast, *HDAC7* expression in REH cells was not altered. HDAC7 protein expression was also upregulated after treatment of SEM-K2 and RS4;11 cells with MI-538 + chidamide (Fig. 4D). Additionally, we confirmed that both MI-463 and MI-503 triggered HDAC7 expression (Supplementary Fig. S6B), underlining again the effect of simultaneously inhibiting Menin-1 and class I HDACs. Instead, no upregulation of HDAC7 was observed in *KMT2A* germline REH cells (Fig. 4E). Remarkably, previously reported HDAC7 targets such as *MMP9* and *CD86* were robustly upregulated, similar to the effect of forced HDAC7 expression [26] (Supplementary Fig. S6C), reinforcing the idea that HDAC7 induction is associated with changes in overall t(4;11) pro-B-ALL cells transcriptome.

In order to verify that the effect of MI-538 and chidamide on t(4;11) B-ALL cells depends on HDAC7 presence, we transduced SEM-K2 cells with shRNA sequence targeting *HDAC7* transcript. Despite its low basal levels in these cells, newly generated SEM-K2-sh*HDAC7* cells showed reduced expression of HDAC7 both at mRNA and protein levels, compared to counterparts transduced with a scrambled sequence (Supplementary Fig. S6D and S6E). As a proof-of-concept, after exposing SEM-K2-sh*HDAC7* cells to MI-538 (+ chidamide) therapy, cells were subjected to in vitro assays to verify whether the blockade of leukemogenic properties in t(4;11) pro-B-ALL cells depends on the induction of HDAC7. Both cell viability and colony formation capacity of SEM-K2-sh*HDAC7* cells were reduced to a significantly lesser extent than in SEM-K2-scrambled cells (Supplementary Fig. S6F and Fig. 4F,G). These data indicate that impairing HDAC7 induction prevents our newly defined combinatorial therapy from exerting its full activity, reassuring the hypothesis that its capacity to block leukemic cells proliferation depends on the option to trigger HDAC7 expression.

### The use of MI-538 and chidamide shifts t(4;11) B-ALL cells transcriptome towards a more differentiated stage

**Fig. 5:**
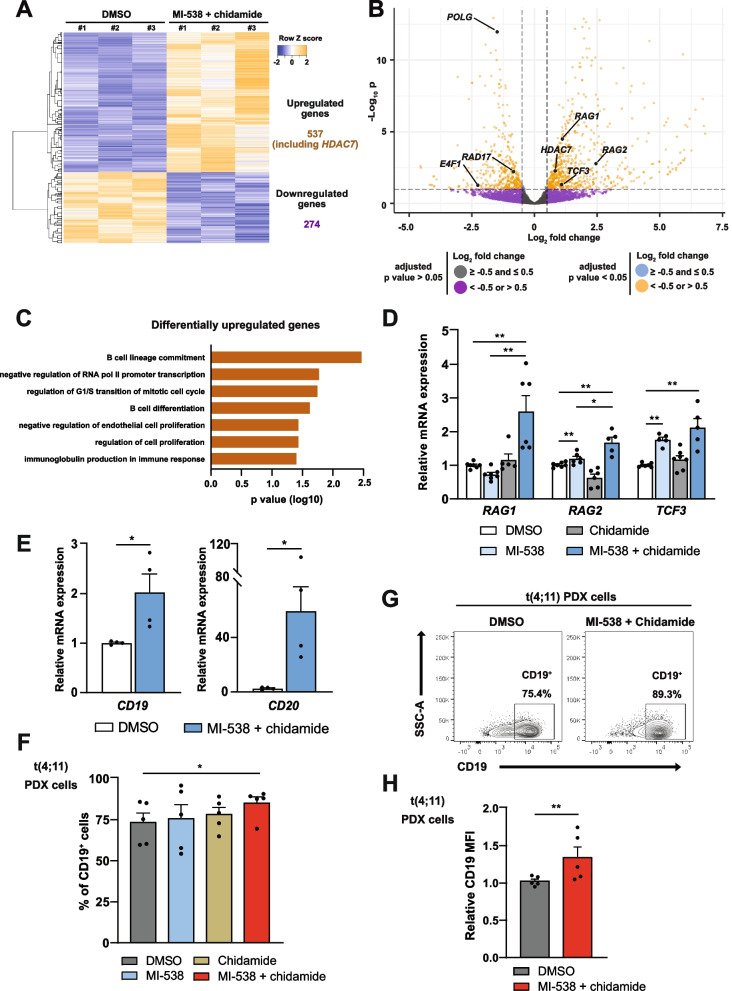
MI-538 and chidamide confer a more differentiated B cell profile to t(4;11) pro-B-ALL cells. **A** Heatmap of the 811 differentially expressed genes (DEGs) identified by comparing bulk RNAseq data from three sets of SEM-K2 cells treated with MI-538 and chidamide with vs SEM-K2 cells treated with DMSO as solvent control. Z-scores are represented from −2 to 2. **B** Volcano plot depicting 811 DEGs shown in (**A**). According to the legend, all DEGs with log2 fold change values < −0.5 or > 0.5 are colored in orange. *HDAC7* and genes of interest for further validation are pointed and highlighted as dark spots. **C** Bar chart representing biological processes (BP) significantly enriched in the set of 537 genes upregulated in (**A**). **D** Expression of *RAG1*, *RAG2* and *TCF3* mRNA by qRT-PCR in SEM-K2 cells treated with 1 µM of MI-538 for 6 days and/or 1 µM of chidamide for 48 h, compared to DMSO as solvent control. *GAPDH* and *RPL38* were used as housekeeping genes (*n* = 5–7 per condition). **E** As in (**D**), but for B lymphocyte markers *CD19* and *CD20*, including only DMSO and MI-538 + chidamide conditions (*n* = 4 per condition). **F** Percentage of CD19^+^ cells in t(4;11) pro-B-ALL PDX samples from patient #1, analyzed by flow cytometry using PE-conjugated anti-human CD19 antibody, after 36-48 h in culture. Where indicated, cells were treated with MI-538 1 µM, chidamide 1 µM or the combination of both drugs (*n* = 5–6 per condition). **G** Representative flow cytometry plot for data in (**F**), including DMSO and MI-538 + chidamide conditions. **H** Mean fluorescence intensity (MFI) of CD19 expression, calculated for CD19^+^ cells gated in (**F**), for DMSO and MI-538 + chidamide conditions (*n* = 5 per condition). Results in (**D**-**F**) and (**H**) are shown as average ± SE. Statistical significance is indicated as: *p < 0.05; **p < 0.01; or n.s. non-significant

Aiming to analyze the biological processes affected by MI-538 + chidamide, we performed RNA-seq analysis of SEM-K2 cells treated with this combinatorial therapy. A total of 811 genes were found to be differentially expressed (Fig. 5A), a gene signature that positively correlated with a previously reported Menin-1 inhibition gene signature (Supplementary Fig. S7A). Of these, 537 genes were upregulated (including *HDAC7*) and 274 were downregulated (Fig. 5A,B). Reassuringly, RNA-seq data confirmed the inverse expression pattern of *HDAC7* and *EZH2* upon MI-538 + chidamide treatment (Supplementary Fig. S7B). Gene ontology analysis revealed that the upregulated genes correlated with the activation of pathways involved in B lymphocyte differentiation and immunoglobulin production, and with cell cycle regulation and proliferation (Fig. 5C). qRT-PCR analysis confirmed also the expression of *RAG1*, *RAG2* and *TCF3* demonstrating that the differentiation block in t(4;11) is relieved [40, 41] (Fig. 5B,D). As a consequence, expression of B lymphocyte differentiation markers (*CD19*, *CD20*) were found upregulated upon combinatorial treatment in SEM-K2 cells (Fig. 5E).

Given the essential role of CD19 in the transition of pro-B cells towards pre-B stage [42], we analyzed whether MI-538 and/or chidamide induce its expression in primary PDX cells with t(4;11) translocation. Importantly, only the combination of both drugs led to an increased CD19^+^ cells percentage ex vivo (Fig. 5F, G), thus reducing the presence of undifferentiated CD19^−^ leukemic cells. Moreover, the shift towards a larger population of CD19^+^ cells was seconded by stronger CD19 expression in PDX cells after MI-538 + chidamide treatment (Fig. 5H). This therapy did not modify CD19 expression in *KMT2A* germline PDX cells (Supplementary Fig. S7C), confirming the specificity of the combined treatment in t(4;11) pro-B-ALL cells.

### The combined use of MI-538 and chidamide improves response of t(4;11) B-ALL cells to glucocorticoid therapy through the modulation of RUNX2 and NR3C1

**Fig. 6:**
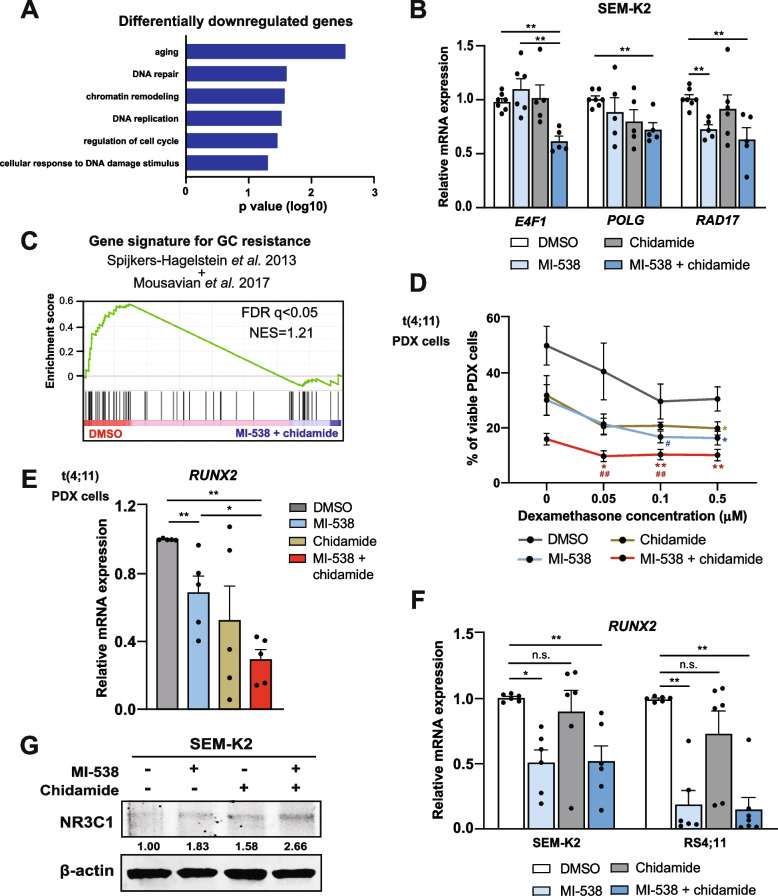
t(4,11) pro-B-ALL cells display increased glucocorticoid sensitivity upon MI-538 + chidamide treatment through the modulation of RUNX2 and NR3C1. **A** Bar chart representing biological processes (BP) significantly enriched in the set of 274 genes downregulated in Fig. 5A. Only pathways related to BP involved in oncogenic processes and including 4 or more genes are included. **B** Expression of *E4F1*, *POLG* and *RAD17* mRNA by qRT-PCR in SEM-K2 cells treated with 1 µM of MI-538 for 6 days and/or 1 µM of chidamide for 48 h, compared to DMSO as solvent control. *GAPDH* and *RPL38* were used as housekeeping genes (*n* = 5–7 per condition). **C** Gene-set enrichment analysis of transcriptomic profiles from SEM-K2 cells treated with MI-538 and chidamide (in blue) of DMSO, as control (in red), from Fig. 5A. GC resistance gene signature combines gene sets from Spijkers-Hagelstein et al*.* 2013 and Mousavian et al*.* 2017. **D** Cell viability assay for pro-B-ALL PDX cells obtained from t(4;11) patients #1 and #2, after 36-48 h in culture. Results are displayed as percentage of Annexin V/7-AAD double negative cells (*n* = 8–9 samples per condition). Where indicated, cells were treated with MI-538 1 µM, chidamide 1 µM or a combination of both drugs. Additionally, samples were treated with increasing dexamethasone concentrations, as labeled. For statistical significance comparisons: *, significance compared to DMSO condition (same concentration of dexamethasone); ^#^, significance compared to DMSO condition with the highest dexamethasone concentration (0.5 µM). **E** Expression of *RUNX2* mRNA by qRT-PCR in t(4;11) pro-B-ALL PDX cells from patients #1 and #2, after 36-48 h in culture. Where indicated, cells were treated with MI-538 1 µM, chidamide 1 µM or the combination of both drugs (*n* = 5 per condition), or DMSO as solvent control. *GAPDH* and *RPL38* were used as housekeeping genes (*n* = 5 per condition). **F** As in (**B**), but for *RUNX2* mRNA levels, expression analyzed in SEM-K2 and RS4;11 cells. *GAPDH* and *RPL38* were used as housekeeping genes (*n* = 6 per condition). **G** Protein levels of NR3C1 in SEM-K2 cells treated with MI-538 (1 µM, 6 days) and/or chidamide (1 µM, 48 h), or DMSO solvent as control. β-actin was used as loading control. Results in (**B**) and (**D**-**F**) are shown as average ± SE. Statistical significance is indicated as: *p < 0.05; **p < 0.01; or n.s. non-significant

The degree of B cell differentiation in lymphoblastic leukemia affects the response of leukemic cells to glucocorticoid (GC) treatment [43]. Moreover, among the set of downregulated genes in Fig. 5A, we found that pathways related to chromatin condensation and DNA repair were enriched (Fig. 6A). Target validation by qRT-PCR confirmed that *E4F1*, *POLG* and *RAD17* (involved in cell cycle regulation, DNA replication and DNA damage response, respectively) [44–46] were repressed upon MI-538 + chidamide treatment (Figs. 5B and 6B). Since the inhibition of DNA damage response is also linked to chemotherapy sensitization [47], we next sought to investigate the effect of MI-538 + chidamide on leukemic cells response to GC therapy.

Interrogation of published GC resistance signatures in the transcriptome from Fig. 5A revealed significant enrichment of these signatures in DMSO-treated cells compared with MI-538 + chidamide-treated SEM-K2 cells (Fig. 6C). Thus, we analyzed the effect of MI-538 and chidamide on the response of leukemic cells to conventional GC therapy. t(4;11) PDX cells were exposed to increasing doses of dexamethasone in the presence of MI-538 and chidamide (either as single agents or in combination) and compared the response with DMSO-treated counterparts. Dexamethasone treatment resulted in significant decrease in cell viability when cells were co-treated with MI-538 and chidamide, even at low doses as 0.05 µM (Fig. 6D). Interestingly, leukemic cells treated with MI-538 and chidamide displayed reduced viability than DMSO-treated cells, even when exposed to a 10-time lower dexamethasone dose, suggesting that this combined therapy may improve GC-resistant patients’ response to standard front-line treatment.

Aiming to decipher the mechanism beyond this increased GC sensitivity, we investigated the expression of transcription factor *RUNX2* in t(4;11) PDX cells treated with MI-538 and/or chidamide. RUNX2, which is included in the gene signature of Fig. 6C, promotes chemotherapy resistance in several types of cancer [48] and inhibits the expression of *NR3C1* (encoding for Glucocorticoid Receptor protein) [49]. As expected, *RUNX2* was repressed by MI-538 + chidamide combination, both in t(4;11) primary cells and SEM-K2 and RS4;11 cell lines (Fig. 6E, F). Opposite to *RUNX2*, we found that MI-538 + chidamide combination releases NR3C1 expression both at mRNA (Supplementary Fig. S7D) and protein levels (Fig. 6G) in SEM-K2 cells.

### MI-538 and chidamide reduce leukemogenic capacity of primary t(4;11) pro-B-ALL cells

**Fig. 7:**
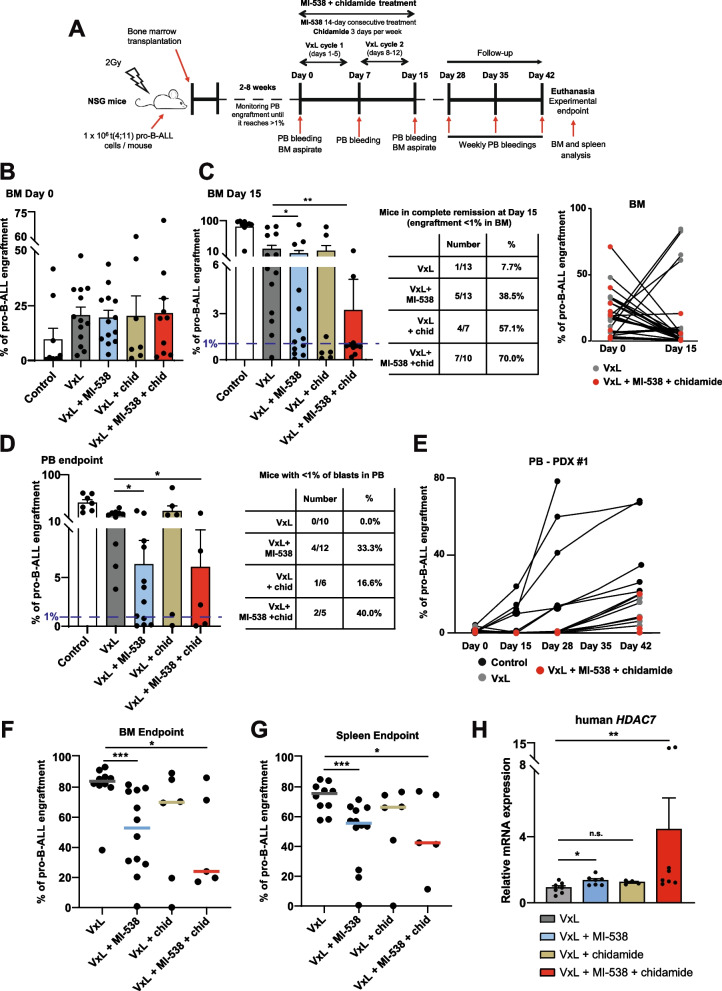
MI-538 and chidamide reduce leukemogenic capacity of primary t(4;11) pro-B-ALL cells. **A** Experimental design of in vivo leukemogenesis assays performed in immunodeficient NSG mice to compare the effect of adding MI-538 and/or chidamide to a conventional chemotherapy regimen (VxL) *vs* VxL treatment alone. **B** Percentage of pro-B-ALL cells engraftment in BM aspirates at Day 0, before starting the treatment. Mice were allocated in the following treatment groups to ensure no statistical differences among them before the treatment: control (*n* = 9), VxL (*n* = 13), MI-538 (*n* = 13), chidamide (*n* = 7) and MI-538 + chidamide (*n* = 10). PDX cells from both t(4;11) pro-B-ALL #1 and #2 were used. **C** Percentage of pro-B-ALL cells engraftment in BM aspirates at Day 15, after treatment completion. *Left panel*, percentage of pro-B-ALL cells engraftment in BM aspirates in all treatment groups. Results shown as average ± SE. *Center panel*, table showing the proportion of mice reaching complete BM remission at Day 15 in each of the groups. *Right panel*, evolution of BM engraftment from Day 0 to Day 15 in VxL (grey dots) and VxL + MI-538 + chidamide (red dots) treatment groups. **D** *Left panel*, average engraftment of t(4;11) pro-B-ALL PDX from patients #1 and #2 in PB at corresponding endpoints in all four treatment groups (*n* = 5–12 per group). *Right panel*, table showing the proportion of mice with undetectable leukemia at Day 42 in each of the groups. **E** Evolution of t(4;11) pro-B-ALL cells engraftment in PB from Day 0 (before treatment) to Day 42 (endpoint) for control (black dots), VxL (grey dots) and VxL + MI-538 + chidamide (red dots) treatment groups. Only PDX cells from t(4;11) pro-B-ALL #1 were used for this comparison. **F** Median BM engraftment of t(4;11) pro-B-ALL PDX cells in all four treatment groups (*n* = 5–12 per group) at experimental endpoint. **G** As in (**F**), but for spleen instead of BM. **H** Expression of human *HDAC7* by qRT-PCR in total BM RNA obtained at experimental endpoint from mice treated with either VxL, VxL + MI-538, VxL + chidamide or VxL + MI-538 + chidamide, as indicated (*n* = 5–8 per group). *GAPDH* and *RPL38* were used as housekeeping genes. Statistical significance is indicated as: *p < 0.05; **p < 0.01; ***p < 0.001; or n.s. non-significant

As proof-of-concept, we sought to validate our data in vivo using two t(4;11) PDX samples to determine whether MI-538 + chidamide treatment improves the response to standard chemotherapy. To this end, PDX cells were transplanted intratibially into sub-lethally irradiated NSG mice, which were later treated either with a conventional chemotherapy regimen (vincristine, dexamethasone and L-asparaginase, VxL), or in combination with MI-538 + chidamide (Fig. 7A), as previously described [50, 51]. Prior to treatment initiation, mice were divided into four different treatment groups with similar BM engraftment (Fig. 7B). Given that growth rate of PDX cells in NSG mice had been previously tested, mice with the lowest BM engraftment were allocated to the untreated group, to confirm proper engraftment of leukemic cells. For all treated mice, analysis of BM aspirates at treatment completion (day 15) showed that 70% of mice in the VxL + MI-538 + chidamide group achieved complete remission in BM, compared with only 7.7% of animals in the VxL group (Fig. 7C). The average of total engraftment detected in BM at this time point showed a significant reduction upon MI-538 addition (alone or plus chidamide), whereas chidamide alone did not show significant differences (Fig. 7C).

For relapse rate determination, mice were followed up to 4 weeks after treatment. Notably, 40% of mice treated with VxL + MI-538 + chidamide had undetectable levels of leukemic B-ALL cells (< 1%) in PB at the end of the protocol, while none of the mice in the VxL group had undetectable levels. Notably, in one-third of mice in the VxL + MI-538 group, leukemic B-ALL cells remained undetectable in PB (Fig. 7D,E and Supplementary Fig. S8A). Similarly, a lower engraftment in the BM and spleen was observed in mice treated with the combinatorial therapy and VxL, compared with counterparts treated with VxL alone. Again, the inclusion of MI-538 alone proved to be significantly beneficial to reduce engraftment (Fig. 7F,G). Spleen weight also revealed a significant reduction in the animals treated with VxL + MI-538 + chidamide (Supplementary Fig. S8B). Importantly, analysis of BM cells extracted at the experimental endpoint revealed that MI-538 + chidamide treatment induced HDAC7 expression in PDX cells (Fig. 7H and Supplementary Fig. S8C), supporting our initial hypothesis that the precise upregulation of HDAC7 reduces the malignant capacity and treatment unresponsiveness of t(4;11) pro-B-ALL.

## Discussion

**Fig. 8:**
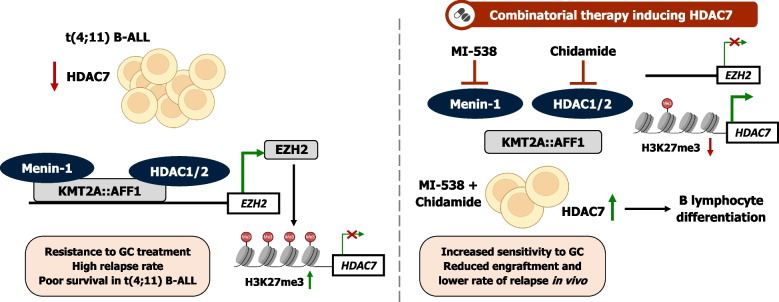
Graphical abstract summarizing the effect of HDAC7 induction in t(4;11) pro-B-ALL. Precise induction of HDAC7 expression in KMT2A::AFF1 pro-B-ALL cells reduces leukemogenesis and delays relapse onset by conferring leukemic cells a more differentiated B cell profile and higher sensitivity to GC treatment

Our findings demonstrate the efficacy of rational therapeutic approaches for high-risk infant B-ALL with the t(4;11) rearrangement. Based on our data, EZH2 and KMT2A::AFF1 are both mediating HDAC7 repression. Given the inability of EZH2 inhibitors to reduce leukemic cells growth, we endeavored to interfere with KMT2A::AFF1 activity and identified a combinatorial therapy of Menin-1 inhibitor (MI-538) in combination with class I HDAC inhibitor (chidamide). Remarkably, this therapy imparted to t(4;11) leukemic cells a greater ex vivo sensitivity to GC, an important key of regular chemotherapeutic regimens. Thus, the here present combination of specific inhibitors demonstrated a significant improvement in terms of leukemia remission and relapse in vivo when added to standard chemotherapy in the future (Fig. 8).

Previous reports have defined HDAC7 as a pro-leukemogenic factor [52–54]. However, these studies were not restricted to infants and included patients with B-ALL of distinct cell origin, including pre-B-ALL. Rather, HDAC7 is a biomarker of favorable prognosis for infants with high-risk t(4;11) pro-B-ALL, and its overexpression confers a leukemia-suppressing phenotype in this set of patients. In fact, the exogenous overexpression of HDAC7 in t(4;11) pro-B-ALL reduces proliferative capacity of leukemic cells, through apoptosis induction. Moreover, chemotherapy resistance markers are depleted upon HDAC7 induction [26]. Accordingly, we show here that t(4;11) pro-B-ALL cells with higher levels of HDAC7 are more likely to behave as germline *KMT2A*, supporting our hypothesis that restoring HDAC7 expression may increase infant patient survival.

An interesting finding underlying HDAC7 downregulation in t(4;11) cells is the increased methylation of H3K27 at the *HDAC7* promoter. These data may appear as contradictory to the concomitant CpG island hypomethylation; however, bivalent epigenetic regulation has been previously reported in embryonic pluripotent cells [28, 55]. Indeed, DNA methyltransferases (DNMTs) and PRC2 members, including EZH2, interact to stabilize gene silencing [56]. Given the origin of infant t(4;11) B-ALL, it is plausible that promoter regions of key genes such as *HDAC7* are regulated by a bivalent chromatin state similarly to embryonic pluripotent cells.

With reference to the repressive effect exerted by EZH2 on *HDAC7* promoter, we show here that t(4;11) pro-B-ALL infants with strongest *EZH2* expression display negligible *HDAC7* levels. However, it is worth noting that the absence of EZH2 in the subgroup of KMT2A::AFF1 cases does not fully trigger *HDAC7* to similar expression than *KMT2A* germline infants. This effect suggests a potential compensatory effect by other PRC2 members, such as EZH1 [57].

In this sense, EZH2 inhibitors were discarded as potential therapeutic strategy since they drive FLT3 signaling induction, thus failing to block t(4;11) pro-B-ALL cells proliferation. This effect had been previously reported in the context of T-ALL [38] and, interestingly, is not shared in *KMT2A*-rearranged leukemias by Menin-1 inhibitors, which repress FLT3 expression by inhibiting transcriptional activators such as MEIS1 [58].

In connection with these data, KMT2A::AFF1 fusion protein promotes leukemia progression mainly by activating pro-leukemogenic factors HOXA9 and MEIS1 [59]. However, the importance of KMT2A::AFF1 in provoking extremely poor outcomes is not limited to oncogene induction. As shown here, it also mediates HDAC7 repression, through an alternative indirect mechanism involving EZH2. This is in accord with previous evidence demonstrating that KMT2A::AFF1 imposes a B-cell lineage block at the pro-B cell stage, whereas fusion proteins derived from other rearrangements block the commitment of B-cell precursors at a later pre-B stage [60]. In parallel, a conditional *Hdac7*-deficient mouse model confirmed that HDAC7 deficiency impairs pro-B to pre-B transition [23, 24]. Collectively, these findings confirm that lack of HDAC7 maintains the leukemogenic phenotype by preventing pro-B cells from transitioning towards a pre-B stage. The blockade of B lymphocyte differentiation at an earlier stage may also contribute to the increased resistance of t(4;11) pro-B-ALL to GC-based chemotherapy [43].

The diversity of partners that bind to KMT2A::AFF1 protein, makes this oncogenic driver a complex target for precision medicine [4]. However, drug candidates are available that block its binding to co-activators, such as DOT1L, class I HDACs or Menin-1 [39, 61–64]. MIs have demonstrated activity in *KMT2A*-rearranged AML [65–67], interfering with tumor suppressor mechanisms such as DNA repair and DNA damage checkpoints [66]. The ability to downregulate DNA damage response, together with the inhibition of GC resistance-associated transcription factor RUNX2 [48], sets Menin-1 inhibition as an efficient strategy in infant B-ALL.

Importantly, the MI revumenib was approved by FDA for the treatment of KMT2A:AFF1-rearranged leukemia before our study was completed. In fact, this MI was shown to induce clinical response in patients with AML with either *NPM1* or *KMT2A* alterations [67]. However, already identified somatic mutations in the *MEN1* gene confer resistance to MI [68]. These mutant clones show a structural impairment in the natural interaction between the MI and KMT2A::AAFF1 and may become dominant upon continuous drug exposure due to selective pressure. In parallel, it was demonstrated that specific inhibition of class I HDACs impairs binding of KMT2A::AFF1 protein to key target genes (such as *ALOX5* locus), while recruiting endogenous wild-type KMT2A [39]. Despite lacking experimental data on this point, we propose that combining MI-538 with chidamide may lead to a partial replacing of KMT2A::AFF1 fusion protein by germline KMT2A to key targets, potentially preventing the emergence of therapeutic resistance [39].

Patients harboring KMT2A rearrangements often activate lineage switch mechanisms, mediated by CD19 loss [69], whereby malignant blasts “hide” common lymphoid markers and acquire myeloid gene expression signatures [70, 71]. Interestingly, Lamble et al. have recently shown that CD19 CAR-T infusion in infants (median age of 0.6 years at diagnosis) provides equivalent rates of complete remission, when compared to older patients [72]. However, this study also demonstrates that the presence of KMT2A rearrangements is the only factor significantly associated to the development of lineage switch which, in turn, is associated to a dismal prognosis after relapse [72]. Our results show that precise HDAC7 induction confers a more differentiated B cell phenotype, including the upregulation of classical B cell markers such as *CD19* and *CD20*. In fact, the use of MI-538 and chidamide reduces the population of leukemic cells lacking CD19 expression, which are more likely to undergo lineage switch and escape from CD19-directed immunotherapy. Accordingly, the therapy proposed may increase the sensitivity of t(4;11) leukemic cells to CAR-T strategies.

In conclusion, we propose that the combinatorial therapy presented here, with the ability to induce HDAC7 biomarker expression, is a promising strategy to improve the response of t(4;11) pro-B-ALL and ultimately improve overall survival. As infants are normally excluded from clinical trials due to their vulnerability, this treatment opens a new field for personalized medicine in infant leukemia.

## Supplementary Information

Supplementary Material 1.

Supplementary Material 2.

## Data Availability

RNA sequencing data is publicly available in GEO repository (GSE268574). Data from DNA methylation arrays is deposited and available in ArrayExpress under E-MTAB-8505 accession number. Processed data was obtained from the OpenAIRE repository Zenodo (10.5281/zenodo.3695639). Processed data for ChIP sequencing analysis have been deposited in the OpenAIRE Zenodo repository (10.5281/zenodo.11550906).

## Abbreviations

B-ALL: B-cell acute lymphoblastic leukemia
HDAC7: Histone deacetylase 7
KMT2A: Lysine Methyltransferase 2A
AFF1: ALF Transcription Elongation Factor 1
EZH2: Enhancer Of Zeste 2 Polycomb Repressive Complex 2
MI: Menin-1 Inhibitor
PDX: Patient-derived Xenograft
GC: Glucocorticoid
VxL: Vincristine, dexamethasone and L-asparaginase
