# Supplementary material for: HDAC7 induction combined with standard-of-care chemotherapy provides a therapeutic advantage in t(4;11) infant B-cell acute lymphoblastic leukemia
Source: Biomark Res. 2025 Jul 28;13:99. doi: 10.1186/s40364-025-00810-1 (PMC12305908; doi:10.1186/s40364-025-00810-1)

Supplementary Figure S1

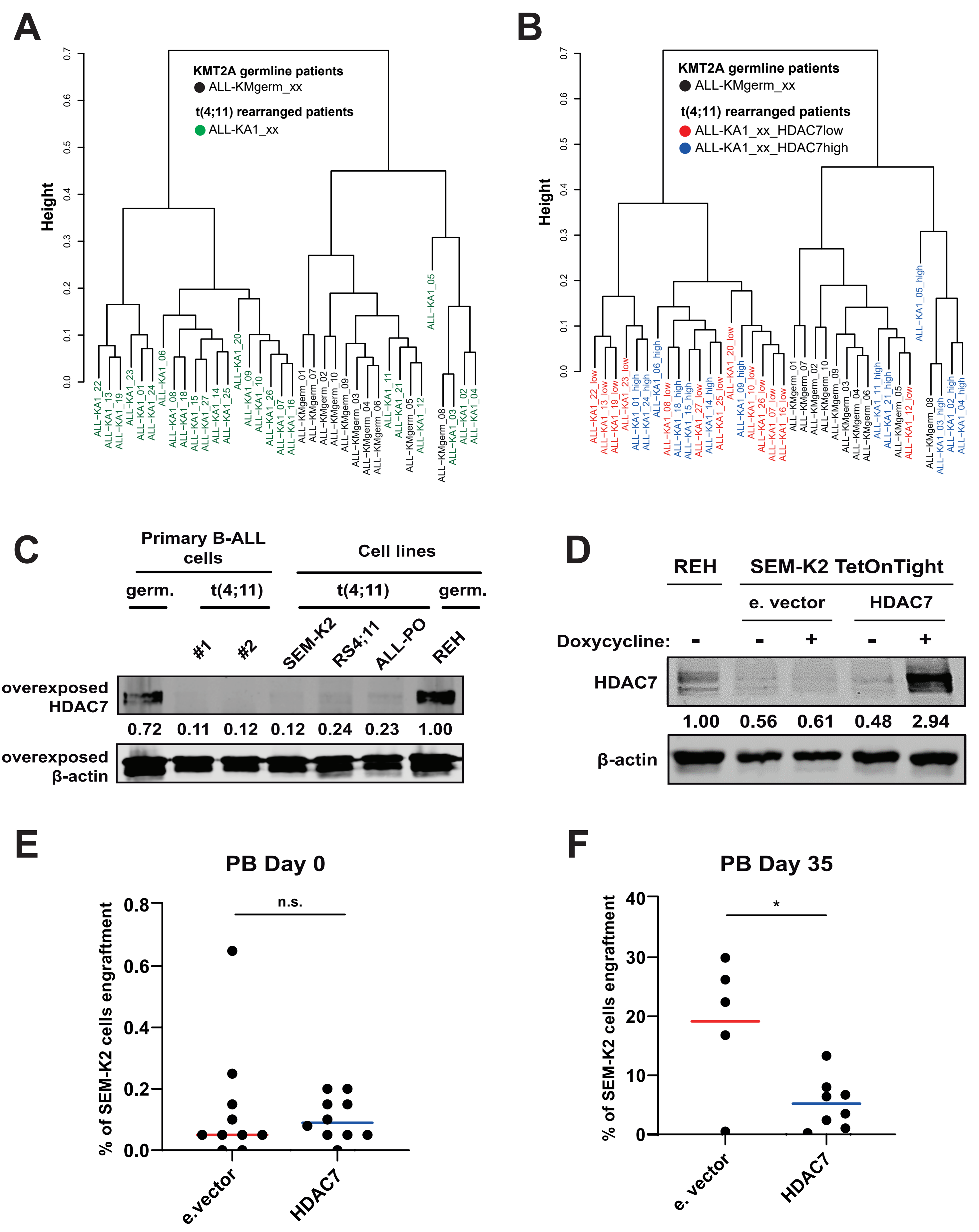

Supplementary Figure S2

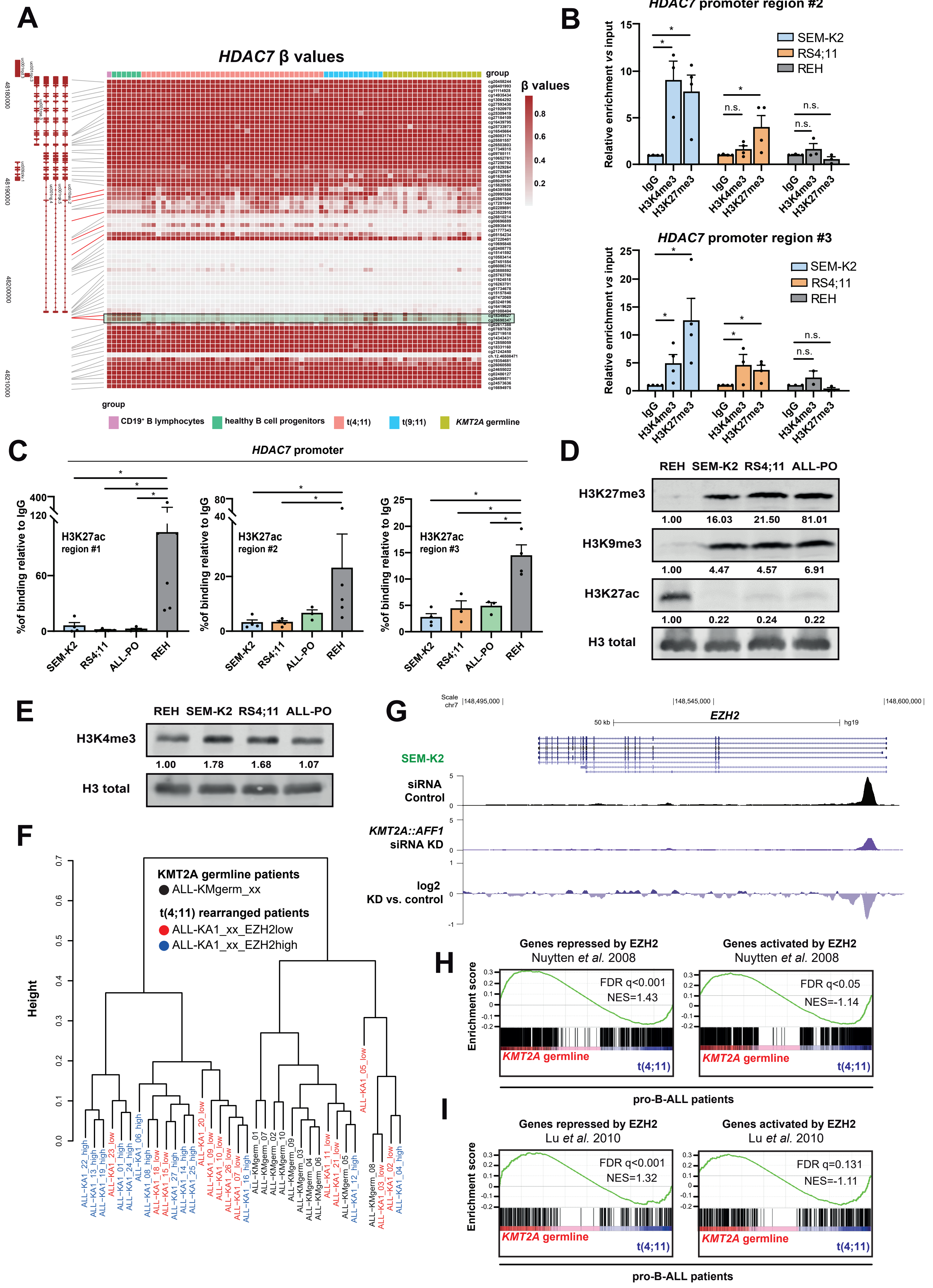

Supplementary Figure S3

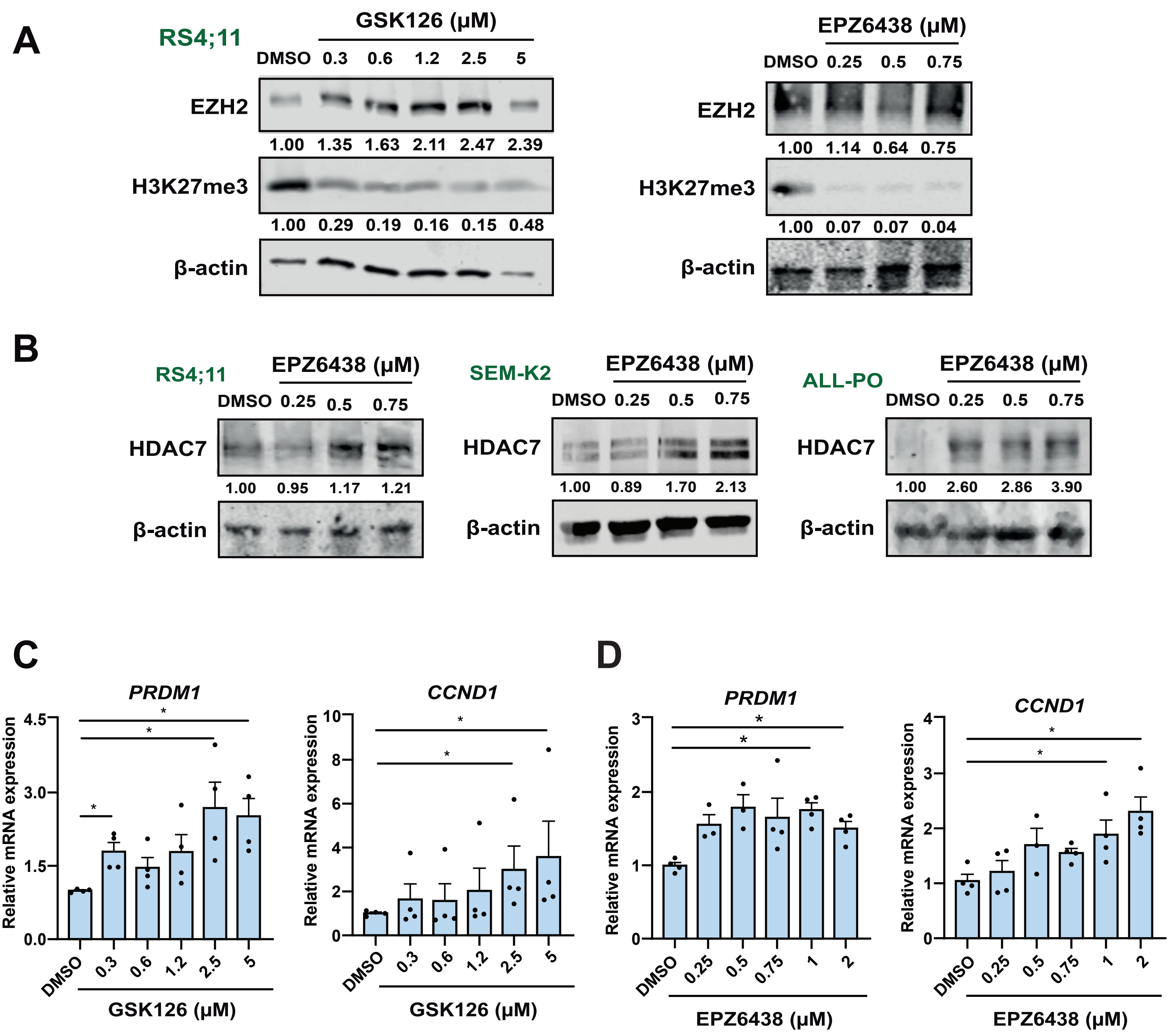

Supplementary Figure S4

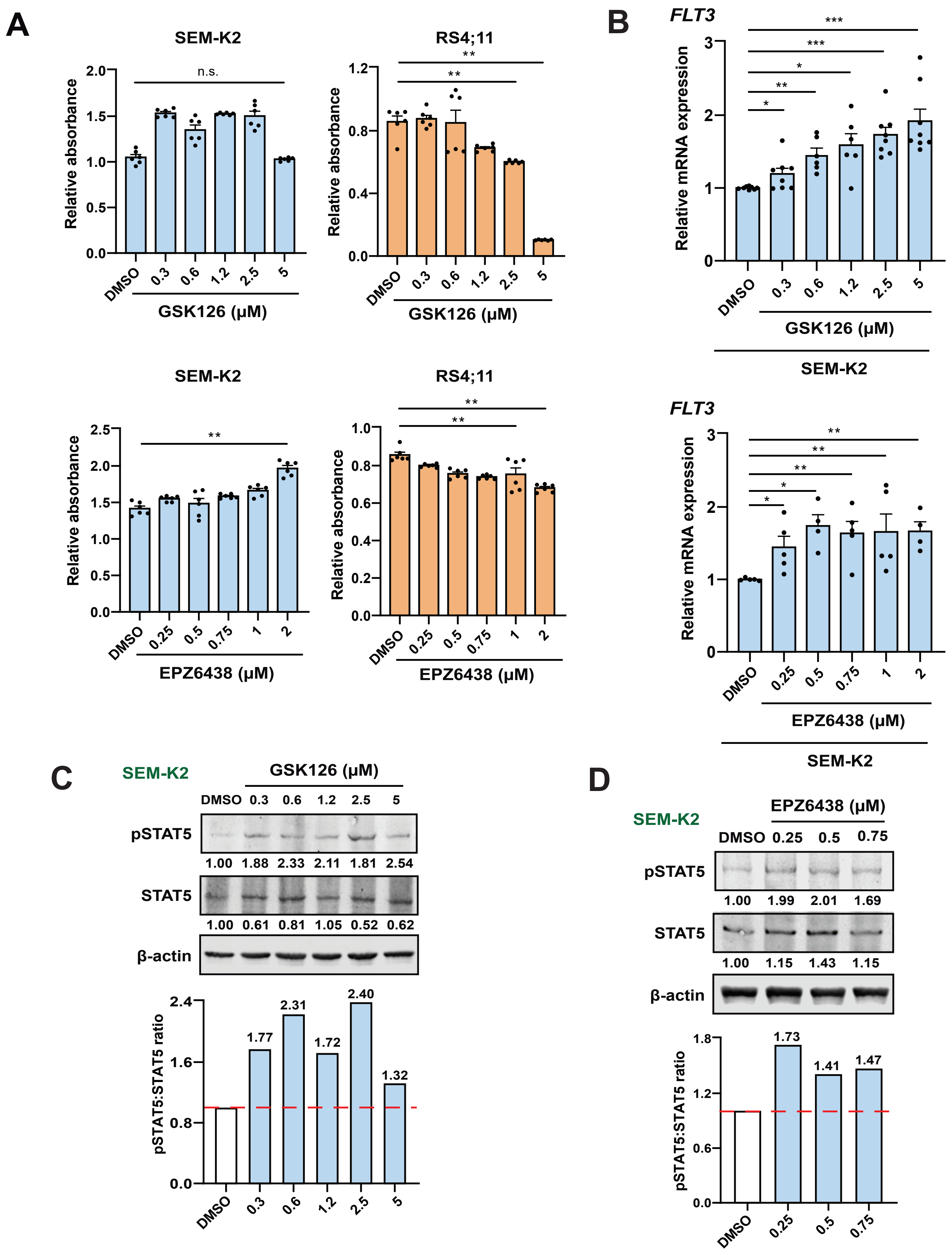

Supplementary Figure S5

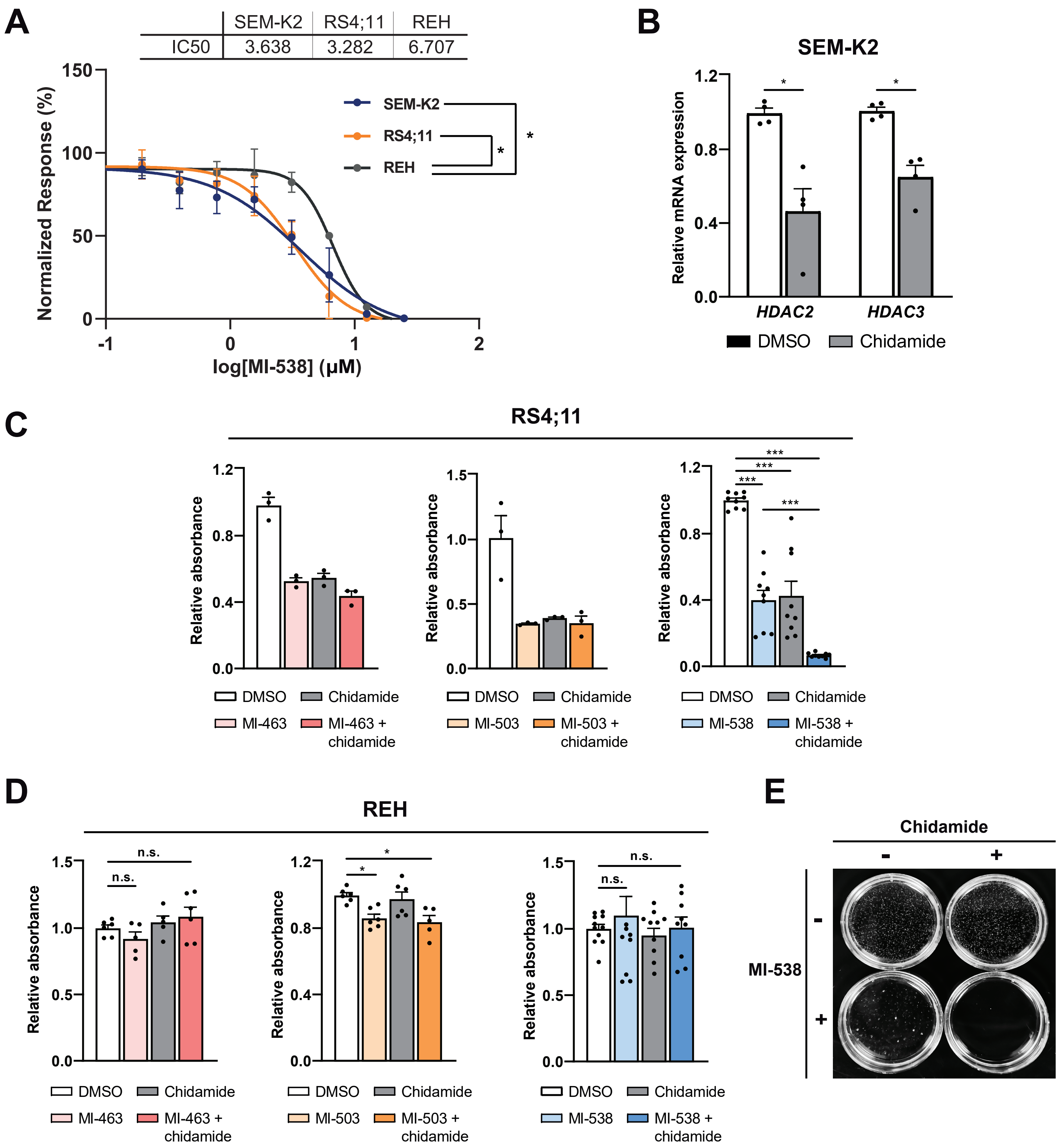

Supplementary Figure S6

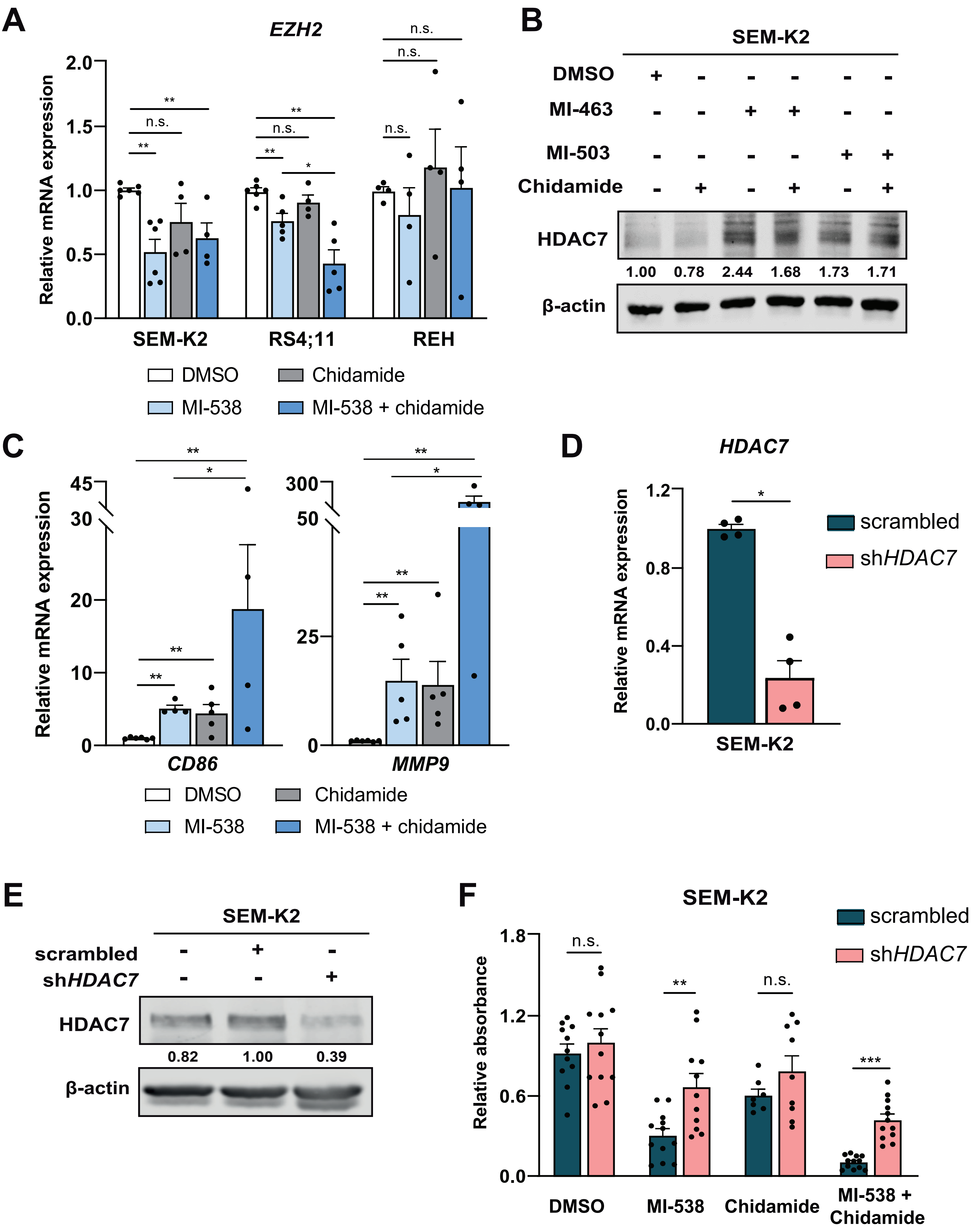

Supplementary Figure S7

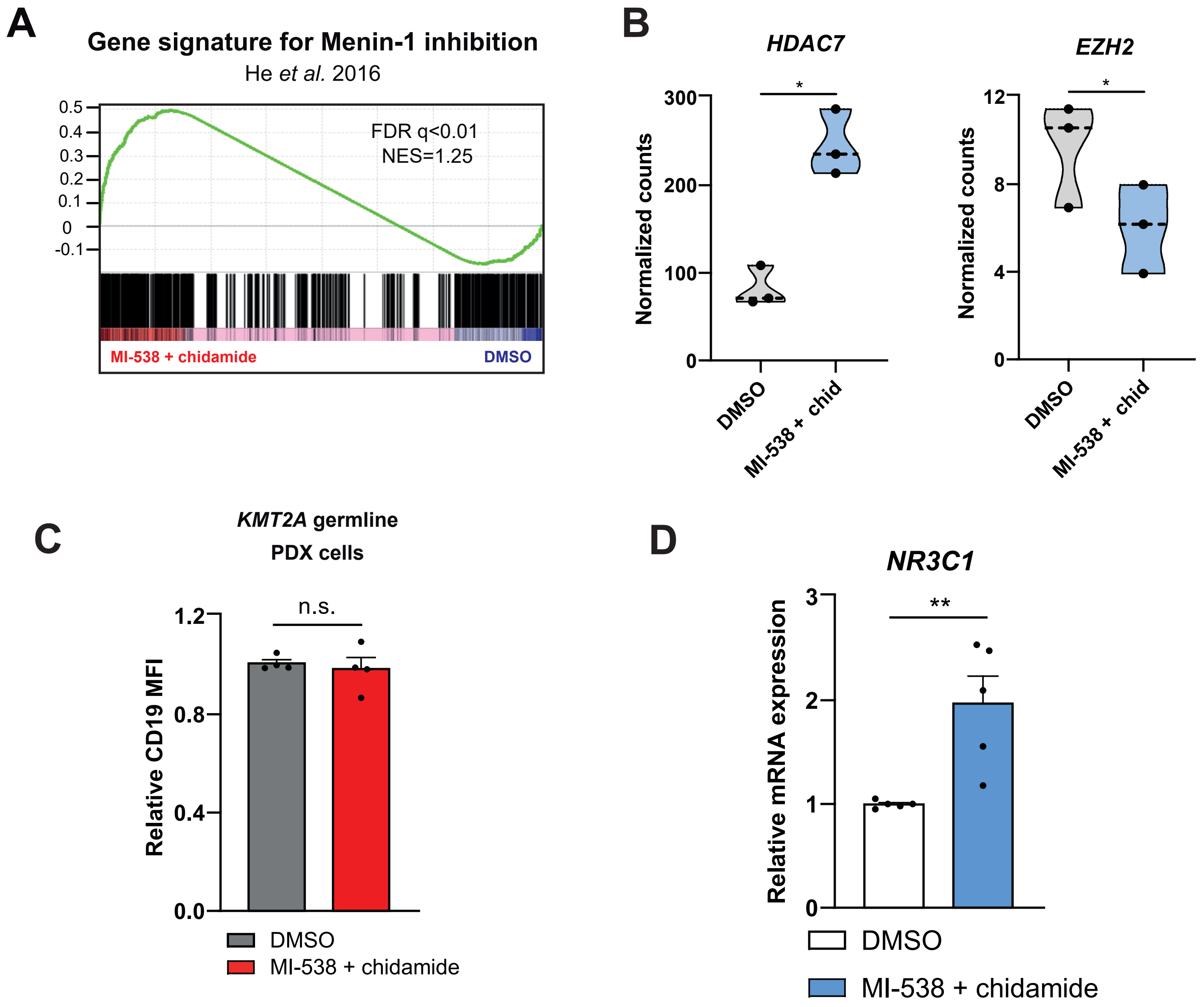

Supplementary Figure S8

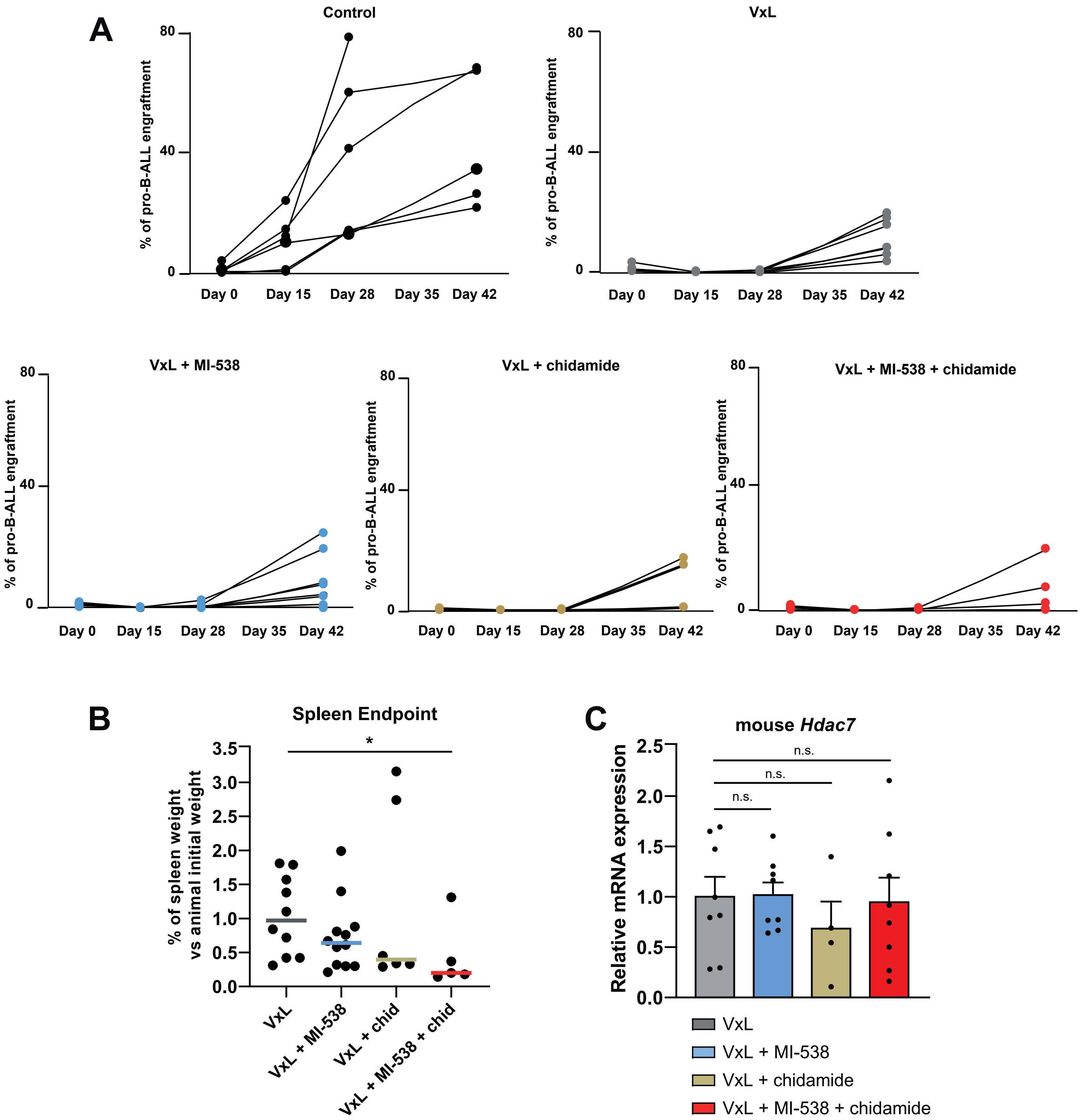

Supplement: Supplementary file 1 — Supplementary Material 1. [file 40364_2025_810_MOESM1_ESM.pdf]
