# Supplementary material for: HDAC7 induction combined with standard-of-care chemotherapy provides a therapeutic advantage in t(4;11) infant B-cell acute lymphoblastic leukemia
Source: Biomark Res. 2025 Jul 28;13:99. doi: 10.1186/s40364-025-00810-1 (PMC12305908; doi:10.1186/s40364-025-00810-1)
Supplement: Supplementary file 2 — Supplementary Material 2. [file 40364_2025_810_MOESM2_ESM.docx]

**Supplementary information**

**for**

**HDAC7 induction combined with standard-of-care chemotherapy provides a therapeutic advantage in t(4;11) infant B-cell acute lymphoblastic leukemia**

**Oriol de Barrios^1^**^*^**, Ingrid Ocón-Gabarró^1,2#^, Mar Gusi-Vives^1,2#^, Olga Collazo^1#^, Ainara Meler^1,3^, Paola A Romecín^4^, Alba Martínez-Moreno^4^, Juan Ramón Tejedor^5,6^, Mario F. Fraga^5,6^, Pauline Schneider^7^, Michela Bardini^8^, Giovanni Cazzaniga^8,9^, Rolf Marschalek^10^, Ronald W Stam^7^, Clara Bueno^4^, Pablo Menéndez^4,11,12,13,14^, Maribel Parra^1*^**

^1^Lymphocyte Development and Disease Group, Josep Carreras Leukaemia Research Institute, 08916 Badalona, Spain

^2^Doctoral Program in Biomedicine, Universitat de Barcelona (UB), Barcelona, Spain

^3^Center for Immuno-Oncology, Center for Cancer Research, National Cancer Institute, Bethesda, Maryland, USA

^4^Josep Carreras Leukaemia Research Institute, School of Medicine, University of Barcelona, 08036, Barcelona, Spain

^5^Nanomaterials and Nanotechnology Research Center (CINN-CSIC), Health Research Institute of Asturias (ISPA), Institute of Oncology of Asturias (IUOPA), 33012, Oviedo, Spain

^6^CIBER-ER-ISCIII, Madrid. Spain

^7^Princess Maxima Center for Paediatric Oncology, Utrecht, The Netherlands

^8^Tettamanti Center, Fondazione IRCCS San Gerardo dei Tintori, Monza, Italy.

^9^School of Medicine and Surgery, University of Milano-Bicocca, Milan, Italy.

^10^Institute of Pharmaceutical Biology/DCAL, Goethe-University, Frankfurt, Germany

^11^Red Española de Terapias Avanzadas (TERAV), Instituto de Salud Carlos III (ISCIII), Madrid, Spain

^12^Department of Biomedicine, School of Medicine, University of Barcelona, Barcelona, Spain

^13^Institució Catalana de Recerca i Estudis Avançats (ICREA), Barcelona. Spain

^14^Centro de Investigación Biomédica en Red (CIBERONC), ISCIII, Madrid, Spain

^#^These authors contributed equally to this work

*Correspondence to:

Dr. Maribel Parra

Josep Carreras Leukaemia Research Institute (IJC)

Ctra de Can Ruti, Camí de les Escoles, s/n

08916 Badalona, Barcelona, Spain,

Phone: +34 935572800

E-mail: [mparra@carrerasresearch.org](mailto:mparra@carrerasresearch.org)

Dr. Oriol de Barrios

Josep Carreras Leukaemia Research Institute (IJC)

Ctra de Can Ruti, Camí de les Escoles, s/n

08916 Badalona, Barcelona, Spain,

Phone: +34 935572800

E-mail:[odebarrios@carrerasresearch.org](mailto:odebarrios@carrerasresearch.org)

**SUPPLEMENTARY METHODS**

**Reagents, drugs and antibodies**

RPMI-1640, IMDM, L-glutamine, penicillin/streptomycin (P/S), and insulin-transferrin-selenium (ITS) were purchased from GIBCO/Invitrogen (Waltham, MA, USA). Fetal bovine serum (FBS) was purchased from Sigma-Aldrich (St. Louis, MO, USA). StemSpan serum-free expansion medium (SFEM) and Methocult H4100 methylcellulose medium were acquired from STEMCELL Technologies (Vancouver, Canada). Human (h) stem cell factor (SCF), hFMS-like tyrosine kinase 3 ligand (FLT3-L), hIL-3 and hIL-7 were purchased from Miltenyi Biotec (Bergisch Gladbach, Germany). G418, puromycin and 7-AAD viability staining were acquired from ThermoFisher Scientific, whereas doxycycline was purchased from Panreac (Barcelona, Spain). EPZ6438, GSK126, MI-2, MI-463, MI-503, MI-538, MI-3454 and Chidamide were acquired from MedChem Express (Monmouth Junction, NJ, USA). Vincristine and dexamethasone for *in vivo* experiments were acquired from Selleckchem (Houston, TX, USA) and Sigma-Aldrich (St. Louis, MO, USA). L-Asparaginase was kindly provided by Jazz Pharmaceuticals (Dublin, Ireland).

The following antibodies were used for flow cytometry assays: FITC-conjugated anti-human Annexin V and anti-human CD19 (HIB19 clone), PE-conjugated anti-human CD19 (HIB19 clone), APC-conjugated anti-human CD45 (HI30) and BV421-conjugated anti-human HLA-ABC (G46-2.6) were acquired from BD Biosciences (Franklin Lakes, NJ, USA). For western blot and chromatin immunoprecipitation assays, the antibodies used were: rabbit anti-human HDAC7 (H-273 clone, Santa Cruz Biotechnology, Dallas, TX, USA; and D4E1L, Cell Signaling Technology, Danvers, MA, USA), anti-human EZH2, anti-human NR3C1, anti-human STAT5 and anti-human phospho-STAT5 (clones D2C9, D8H2, D3N2B and D47E7, respectively, Cell Signaling Technology); anti-human histone 3 total, anti-human H3K9me3 and anti-human H3K27ac (ab1791, ab8889 and ab4729, respectively; Abcam, Cambridge, UK), anti-human H3K27me3 (07-449, Merck Millipore, Burlington, MA, USA) and anti-human H3K4me3 (17-614, Merck Millipore) and anti-mouse β-actin (AC-15; Sigma-Aldrich, St. Louis, MO, USA).

**Cell cultures and generation of stable cell lines**

Human SEM-K2, RS4;11 and REH cell lines were grown and maintained in regular cell culture conditions (RPMI 1640 medium + 10% fetal bovine serum), whereas ALL-PO cells were grown with 20% fetal bovine serum. All cell lines used had been previously authenticated at Genomics Unit of Germans Trias i Pujol Research Institute (IGTP, Badalona, Spain). SEM-K2 TetOn-Tight-empty vector and SEM-K2 TetOnTight-HDAC7 were cultured in in RPMI 1640 complete medium and selection with G418 (1.5 mg/mL) and puromycin (0.4 µg/mL), as described[1]. Once they were selected, SEM-K2 TetOn-Tight-empty vector and SEM-K2 TetOnTight-HDAC7, were cultured in RPMI 1640 complete medium and selection with G418 (1.5 mg/mL) and puromycin (0.4 µg/mL) was maintained. Where indicated, cells were treated with doxycycline at 1 µg/mL.

In order to generate SEM-K2-sh*HDAC7* stable cell line, cells were infected with pSUPER retroviral vector containing shRNA sequence against HDAC7 mRNA transcript and selected for two weeks with puromycin (0.4 µg/mL). The same procedure was used to obtain SEM-K2-scrambled cells, but using a scrambled sequence instead. Both cell lines were maintained in RPMI 1640 complete medium with puromycin (0.4 µg/mL). The sequences used for the generation of pSUPER vectors are: shHDAC7 (forward: gatccggctataagcccaagaagtcttcaagagagacttcttgggcttatagcttttttacgcgtg; and reverse: aattcacgcgtaaaaaagctataagcccaagaagtctctcttgaagacttcttgggcttatagccg) and scrambled (forward: cctaaggttaagtcgccctcgctcgagcgagggcgacttaaccttagg; and reverse: cctaaggttaagtcgccctcgctcgagcgagggcgacttaaccttagg).

**Patient-derived xenograft (PDX) generation and *ex vivo* cell viability analysis**

For PDX obtention, 1x10^6^ cells cultured in IMDM medium will be transplanted in BM of sublethally irradiated (2 Gy) NSG immunodeficient mice (intratibial injection). Peripheral blood (PB) was analyzed bi-weekly by FACS, using human CD19 (1:100), CD45 (1:100) and HLA-ABC (1:200) markers. When PB engraftment of human blasts reached 15%, mice were euthanized and total cells from BM, spleen and liver harvested for PDX obtention.

For *ex vivo* assays PDX cells from two t(4;11) patients (PDX #1, cells and obtained after diagnosis; and PDX #2, cells obtained after relapse) and one KMT2A germline patient were used. Patient #1 corresponds to ALL-KA1_26 in *Supplementary Figs. S1A* and *S1B*, whereas patient #2 and *KMT2A* germline patient are not included in this dataset. For flow cytometry analysis of cells viability and expression of CD19 surface marker, as well as for mRNA and/or protein analysis, 1 x 10^5^ cells per condition were cultured in StemSpan SFEM medium supplemented with 20% FBS, P/S, ITS, hSCF (100 ng/mL), hFLT3-L (100 ng/mL), hIL-3 (10 ng/mL), and hIL-7 (10 ng/mL) for 36-48h. Where indicated, cells were treated with MI-538 and/or Chidamide (1µM each) or with the corresponding amount of DMSO as control. Moreover, for GC resistance assays, increasing doses of dexamethasone (from 0.05 to 50 µM). For cell viability analysis, cells were washed and stained with anti-human CD19 (1:100), anti-human Annexin V (1:100) and 7-AAD (1:50), for FACS.

***In vivo* leukemia models with cell lines and PDX cells**

For *in vivo* leukemia assays with SEM-K2 TetOn-Tight-empty vector and SEM-K2 TetOn-Tight-HDAC7 cells, 8-12 week-old (NOD) Cg-Prkdc^scid^ Il2rg^tm1Wjl^/SzJ (NSG) mice were sublethally irradiated at a 2 Gy dose. A total of 0.5 x 10^6^ empty vector or HDAC7 cells were then transplanted in BM by intratibial injection in age-matched mice. One week after transplantation, after confirming initial engraftment of SEM-K2 cells in PB, doxycycline was administered in drinking water at 1 mg/mL concentration, with 5% sucrose during 14 days. Engraftment of leukemic cells in PB was analyzed weekly, whereas BM aspirates were only analyzed at Day 15. Engraftment of leukemic cells was measured with human markers CD19, CD45 and HLA-ABC. After doxycycline treatment, mice were followed for five weeks with weekly PB analysis. During this period, health assessment was conducted by both the researchers and the personnel at animal facility. Animals in poor health conditions or reaching PB engraftment > 15% were humanely euthanized. This situation was considered the experimental endpoint for each animal. At Day 35, two mice bearing TetOnTight-HDAC7 cells were euthanized to allow comparison with counterparts injected with TetOnTight empty vector cells. Finally, mice reaching Day 50 of the procedure were humanely euthanized and BM and spleen analyzed for leukemic cells engraftment.

For *in vivo* assays PDX #1 and #2 from t(4;11) patients were used. NSG mice were also employed and a total of 1.0 x 10^6^ cells per mice were transplanted in BM after sublethal irradiation. Next, PB was bi-weekly monitored by FACS, until 1% of leukemic cells engraftment was observed, using the same markers mentioned above. Then, mice were equally distributed in treatment groups according to the PB engraftment, to ensure no basal differences: a) untreated control group; b) VxL (Vincristine 0.15 mg/Kg, Dexamethasone 5 mg/Kg and L-Asparaginase 1000 IU/Kg); c) VxL + MI-538 (45 mg/Kg); d) VxL + Chidamide (10 mg/Kg); and e) VxL + MI-538 + Chidamide (same doses as in groups c and d). The whole treatment protocol lasted for 14 days, with VxL being administered in two 5-day cycles separated by two free days (Vincristine is only administered the initial day of each cycle). When corresponding, MI-538 was administered for 14 consecutive days, while Chidamide was administered three times/week. All drugs were injected intraperitoneally. BM aspirates were analyzed again just after treatment completion (Day 15) and, afterwards, PB was extracted weekly for analysis of potential relapse, during 4 weeks after treatment. At day 42 post-treatment initiation, mice were humanely euthanized and leukemic cells engraftment in BM and spleen were analyzed. Additionally, mRNA was extracted from BM samples for further analysis.

For all mice experiments, sex was not considered a variable and males and females were included in equal proportions in the experiments. The number of mice required for statistical power in *in vivo* assays was calculated with GPower software (University of Düsseldorf, Germany). Per our previous experience in murine leukemia models, we employed the following parameters: expected proportions difference of 0.5; standard deviation expected of 0.2; and confidence level of 95%. With these settings, sample size resulted in 24 animals per comparison.

**RNA extraction and qRT-PCR**

Total RNA from PDX samples, SEM-K2 TetOn-Tight-empty vector and HDAC7, SEM-K2-scrambled, SEM-K2-shHDAC7, as well as SEM-K2, RS4;11 and REH cell lines was extracted using Trizol (Life Technologies, Thermo Fisher), following the manufacturer’s instructions. Total RNA samples from pro-B-ALL patients belong to the same cohort of patients previously published (de Barrios et al. Leukemia 2021). After being quantified, 1-2 μg of RNA were retrotranscribed with random hexamers using a High-Capacity cDNA Reverse Transcription kit (Applied Biosystems, Thermo Fisher). mRNA levels were determined by qRT-PCR using SYBR Green PCR Master Mix (Applied Biosystems) in a QuantStudio 7 Flex Real-Time PCR (Applied Biosystems) apparatus. Results were analyzed taking *GAPDH* and *RPL38* as housekeeping genes. All primer pairs were designed with Primer3^®^ software (RRID:SCR_003139). Primers used for gene expression analysis are listed in **Supplementary Tables S1** and **S2**.

**RNA sequencing**

Total RNA was extracted from SEM-K2 cells treated with MI-538 (1 µM for 6 days) and Chidamide (1 µM for 48 h), or DMSO as control, with RNeasy Mini Column kit (Qiagen) for RNA sequencing experiments. Samples were quantified and subjected to quality control using a Bioanalyzer apparatus (IGTP, Badalona, Spain). Part of the RNA samples were kept to check the correct induction of *HDAC7* in the samples. Low input library was performed in all samples, and then 100bp PE sequenced at Macrogen Genomics (Seoul, South Korea), obtaining a minimum of 60M reads per sample. Quality control of the samples was performed with FASTQC tool (available at <https://www.bioinformatics.babraham.ac.uk/projects/fastqc/>). Paired-end reads were aligned to the murine reference genome (GRCm38) using STAR (version 2.7.0a, RRID:SCR_004463)[2]. A count table file indicating the number of reads per gene in each sample was generated using HTSeq (version 0.10.0, RRID:SCR_005514)[3]. Genes with no or very low expression were filtered out and differentially expressed genes were identified using DESeq2 (RRID:SCR_015687) [4], requiring a minimum adjusted p-value of 0.05 and a |log2FC| value greater than 0.5. Gene set enrichment analysis was performed with DAVID (RRID:SCR_001881) tools[5]. Functional analysis was performed using gene set enrichment analysis (GSEA, RRID:SCR_003199)[6] using pre-ranked lists of curated gene sets or, where indicated, previously published gene signatures[7–9] (available at: <https://www.gsea-msigdb.org/gsea/msigdb/>). RNA sequencing data from *KMT2A* germline and t(4;11) pro-B-ALL patients was obtained from publicly available sources[10], and analyzed following the same pipeline previously detailed. The assessment of gene signatures in available data from patients’ samples was also performed by GSEA.

**Colony formation assays**

For analysis of colony formation capacity, SEM-K2, SEM-K2-scrambled and SEM-K2-sh*HDAC7* cells were treated with MI-538 (1 μM) and/or Chidamide (1 μM) 24h prior to plating the assay, along with control cells treated with DMSO. One thousand cells per dish were plated in IMDM medium (20% FBS, 1% P/S, 1% L-Glutamine) diluted at 1:10 proportion in Methocult H4100 methylcellulose medium and each condition was plated in triplicate. Where appropriate, MI-538 and/or Chidamide were added to the media. Colonies were incubated for up to 14 days (37ºC, 5% CO_2_), and counted micro- and macroscopically every 2-3 days. Number of colonies shown is the average of 4 independent experiments.

**DNA methylation arrays and ChIP sequencing analysis**

Microarray-based DNA methylation profiling was performed with Illumina’s Infinium Human Methylation EPIC 850K beadchip platform, using available data published[11]. Bisulfite conversion of DNA was performed as above. Data is deposited and available in ArrayExpress (RRID:SCR_002964) under E-MTAB-8505 accession number. Processed data was obtained from the OpenAIRE repository Zenodo ([10.5281/zenodo.3695639](https://zenodo.org/doi/10.5281/zenodo.3695639)).

ChIP sequencing tracks were obtained from publicly available GEO datasets. GSE202449, GSE83671, GSE38403 and GSE74812 were used to analyze KMT2A immunoprecipitations in primary pro-B-ALL blasts and CCRF, KOPN, RS4;11 and SEM-K2 cell lines[12–15]. GSE202568 was used to analyze N-terminal KMT2A immunoprecipitation in SEM-K2 cells treated with siRNA against KMT2A (SEM-K2 *KMT2A::AFF1* KD) and their counterpart cells treated with control siRNA (SEM-K2 *KMT2A::AFF1* siRNA Ctrl.)[12]. Data was uniformly processed using the ChIP-seq pipeline from the nf-core framework[16], and sequencing reads mapped to the GRCh19 genome using the default parameters for proper normalization purposes. Bigwig and narrowpeak files resulting from these analyses have been deposited in the OpenAIRE Zenodo repository (10.5281/zenodo.11550906). Data accession numbers for methylation arrays and ChIP sequencing are available in **Supplementary Table S3**.

**Protein extraction and western blot**

For protein extraction, cells were washed with ice-cold PBS and resuspended in RIPA lysis buffer (150 mM NaCl, 50 mM Tris–HCl pH 8.0, 1% NP40, 0.1% SDS, 0.5% sodium deoxycholate) containing Complete Mini EDTA-free protease inhibitors cocktail (Roche), PMSF and DTT. Lysates were sonicated in a UP50H ultrasonic processor (Hielscher), clarified by maximum speed centrifugation and quantified with Bradford reagent (Bio-Rad). Protein lysates were boiled and loaded onto 10% polyacrylamide gels and transferred to 0.2-μm nitrocellulose membranes (Amersham Protran, GE Healthcare Lifescience). After blocking with 5% non-fat milk, membranes were blotted overnight (4ºC) with primary antibodies against HDAC7, EZH2, phosphorylated STAT5, total STAT5, NR3C1, H3K27ac, H3K27me3, H3K9me3, H3K4me3, histone 3 total, and β-actin at 1:1000 dilution, except for HDAC7 clone H-273 and EZH2 clone D2C9, that were used at 1:500 dilution. After washing with TBS-Tween, membranes were incubated with fluorescence-conjugated secondary antibodies at 1:3000 dilution (anti-mouse IRDye 680 RD and anti-rabbit IRDye 800CW; LI-COR, Lincoln, NE, USA) for 1-2 h at r.t. After washing with TBS-Tween, membranes were developed using Odyssey CLx imaging system (LI-COR). Images shown are representative of at least three independent experiments. The quantification values shown are relative to the corresponding loading control in each experiment and was performed with FIJI-ImageJ® software. For each protein analyzed, the control condition was set at 1.00.

**Chromatin immunoprecipitation (ChIP)**

SEM-K2, RS4;11, ALL-PO and REH cell lines were used for ChIP assays. Crosslinking of pelleted cells was performed with formaldehyde 1%, and reaction was stopped after 15 min with glycine 1M. After washing crosslinked pellets, cells were lysed by sonication in a Covaris Focus Ultrasonicator. Sonication time varies between 12 and 20 min according to cell lines. Samples were centrifuged 10 min, at 8ºC and at 13000 rpm and cell debris was discarded. 20 μl of sample were separated as an input at this step. Pre-clearing of samples was performed by incubation with Protein A-Salmon Sperm agarose beads (Merck Millipore). For immunoprecipitation, Protein G beads (Thermo Fisher Scientific) were coated with 4ug of the required antibodies or the corresponding anti-rabbit IgG (Santa Cruz) as control for each condition. Antibodies used were rabbit anti-human EZH2, anti-human H3K4me3, anti-human H3K27me3 and anti-human H3K27ac.

Samples were then incubated with coated beads overnight at 4ºC and shaking. After centrifugation, desired chromatin regions pulled down by the specifically coated Protein G beads, which were then washed. Elution of chromatin from protein G beads was performed with an elution buffer containing 1% SDS and 100mM NaHCO3. NaCl 5M was then added to each sample followed by incubation at 65ºC for 4 hours. After adding EDTA 500 μM, Tris pH 6.5 1M and Proteinase-K, samples were again incubated for 1 hour at 45ºC. For DNA purification, phenol-chloroform was then added to each sample and aqueous phase was collected and incubated overnight at -20ºC with sodic acid and glycogen in absolute ethanol. After a 30-minute centrifuge, supernatant was discarded, and the pellet washed with ethanol and later resuspended in nuclease-free water.

The genomic DNA fragments obtained were subjected to quantitative real-time PCR to analyze expression of specific fragments of human *HDAC7* promoter using primers for 3 different regions: region #1 (forward: agggccactgcctaaagc; reverse: ccggtccctatgagtcaaac; fragment size: 159bp), region #2 (forward: atttcttttggaggcagcat; reverse: agtgcctcctggtacttaggg; 150bp) and region #3 (forward: cagctggagaagggttcttg; reverse: gagtccagctactgccttgg; 127bp).

**MTT cell viability assays**

For MTT assays, 5 x 10^4^ SEM-K2, SEM-K2-scrambled, SEM-K2-shHDAC7, RS4;11 or REH cells were seeded in 12-well plates, in triplicate for each condition tested. At the indicated times (maximum of 6 days with MI treatment), MTT reagent was added at 5 mg/mL. Cells were incubated for 3 h under regular conditions and, afterwards, the resulting formazan blue product solubilized in DMSO. Absorbance was measured as the difference between absorbance at 570 nm and background absorbance at 750 nm, using a MultiScan Sky plate reader (ThermoFisher Scientific). Data are presented as average of at least three independent experiments, each of them performed in triplicate.

**Determination of inhibitory concentration 50 (IC50)**

For determination of IC50 in each cell line employed, 5 x 10^3^ cells per well were plated in 96-well plates and treated with increasing doses of the indicated drugs in 150 μL of final volume. After 72h of incubation, 100 μL of Tris-EDTA SDS lysis buffer (10 mM Tris-HCl pH 8, 1 mM EDT, 0.1% SDS) were added to each well and, after overnight incubation, absorbance was read at 560nm with a MultiScan Sky plate reader. IC50 determination for MI-538 was repeated, at least, 4 times for each cell line. The value shown represents average of all independent replicates, whereas drug-response curve shown is representative from all independent assays.

**SUPPLEMENTARY REFERENCES**

1. de Barrios O, Galaras A, Trincado JL, Azagra A, Collazo O, Meler A, et al. HDAC7 is a major contributor in the pathogenesis of infant t(4;11) proB acute lymphoblastic leukemia. Leukemia 2021;35:2086-91.

2. Dobin A, Davis CA, Schlesinger F, Drenkow J, Zaleski C, Jha S, et al. STAR: ultrafast universal RNA-seq aligner. Bioinformatics 2013;29:15–21.

3. Anders S, Pyl PT, Huber W. HTSeq—a Python framework to work with high-throughput sequencing data. Bioinformatics 2015;31:166–9.

4. Love MI, Huber W, Anders S. Moderated estimation of fold change and dispersion for RNA-seq data with DESeq2. Genome Biol 2014;15:550.

5. Huang DW, Sherman BT, Lempicki RA. Systematic and integrative analysis of large gene lists using DAVID bioinformatics resources. Nat Protoc 2009;4:44–57.

6. Subramanian A, Tamayo P, Mootha VK, Mukherjee S, Ebert BL, Gillette MA, et al. Gene set enrichment analysis: A knowledge-based approach for interpreting genome-wide expression profiles. Proc Natl Acad Sci 2005;102:15545–50.

7. Nuytten M, Beke L, Van Eynde A, Ceulemans H, Beullens M, Van Hummelen P, et al. The transcriptional repressor NIPP1 is an essential player in EZH2-mediated gene silencing. Oncogene 2008;27:1449–60.

8. Lu C, Han HD, Mangala LS, Ali-Fehmi R, Newton CS, Ozbun L, et al. Regulation of Tumor Angiogenesis by EZH2. Cancer Cell 2010;18:185–97.

9. He S, Malik B, Borkin D, Miao H, Shukla S, Kempinska K, et al. Menin-MLL inhibitors block oncogenic transformation by MLL-fusion proteins in a fusion partner-independent manner. Leukemia 2016;30:508–13.

10. Agraz-Doblas A, Bueno C, Bashford-Rogers R, Roy A, Schneider P, Bardini M, et al. Unraveling the cellular origin and clinical prognostic markers of infant B-cell acute lymphoblastic leukemia using genome-wide analysis. Haematologica 2019;104:1176–88.

11. Tejedor JR, Bueno C, Vinyoles M, Petazzi P, Agraz-Doblas A, Cobo I, et al. Integrative methylome-transcriptome analysis unravels cancer cell vulnerabilities in infant MLL-rearranged B cell acute lymphoblastic leukemia. J Clin Invest 2021;131:e138833

12. Crump NT, Smith AL, Godfrey L, Dopico-Fernandez AM, Denny N, Harman JR, et al. MLL-AF4 cooperates with PAF1 and FACT to drive high-density enhancer interactions in leukemia. Nat Commun 2023;14:5208.

13. Kerry J, Godfrey L, Repapi E, Tapia M, Blackledge NP, Ma H, et al. MLL-AF4 Spreading Identifies Binding Sites that Are Distinct from Super-Enhancers and that Govern Sensitivity to DOT1L Inhibition in Leukemia. Cell Rep 2017;18:482–95.

14. Geng H, Brennan S, Milne TA, Chen W-Y, Li Y, Hurtz C, et al. Integrative epigenomic analysis identifies biomarkers and therapeutic targets in adult B-acute lymphoblastic leukemia. Cancer Discov 2012;2:1004–23.

15. Benito JM, Godfrey L, Kojima K, Hogdal L, Wunderlich M, Geng H, et al. MLL-Rearranged Acute Lymphoblastic Leukemias Activate BCL-2 through H3K79 Methylation and Are Sensitive to the BCL-2-Specific Antagonist ABT-199. Cell Rep 2015;13:2715–27.

16. Ewels PA, Peltzer A, Fillinger S, Patel H, Alneberg J, Wilm A, et al. The nf-core framework for community-curated bioinformatics pipelines. Nat Biotechnol 2020;38:276–8.

**SUPPLEMENTARY TABLE S1**

Human DNA primers used for quantitative real-time PCR

| **Human**  **gene** | **Forward 5’ – 3’** | **Reverse 5’ – 3’** | **Amplicon size (bp)** |
| --- | --- | --- | --- |
| *RPL38* | TGGGTGAGAAAGGTCCTGGTCCG | CGTCGGGCTGTGAGCAGGAA | 100 |
| *GAPDH* | TCTTCTTTTGCGTCGCCAG | AGCCCCAGCCTTCTCCA | 171 |
| *HDAC7* | CACAGCGGATGTTTGTGATG | TCACGAGAAGCCACTTTGAA | 142 |
| *EZH2* | ACAGTGATAGGGAAGCAGGG | ACCACTCCACTCCACATTCT | 166 |
| *E4F1* | CCACGCATCTGACCTTGTTG | TGTTCACCAGTAGCTTCACCT | 167 |
| *POLG* | GAGCAGCAGACCGGGAAG | CCACCTTCCTCCAGAGCAG | 209 |
| *RAD17* | ACTGGAAATGAAGACCTGCA | CTGTAATAGTTCCCATCCGTAGT | 156 |
| *RAG1* | GTTGGAATGAGGATGGCGAC | AAGGATCTCACCCGGAACAG | 178 |
| *RAG2* | GGCAAAGATTCAGAGAGGCG | CAGGATCTTTTGGGCCAGC | 192 |
| *TCF3* | GACGCACCTTTGTCCGGG | GGTCACTGAGCTCCTTGTCT | 178 |
| *PRDM1* | TACCCTTATCCCGGAGAGCT | CTTTGGAGGGGTTGGAGTCC | 159 |
| *CCND1* | ACAGATCATCCGCAAACACG | GACAGGAAGTTGTTGGGGCT | 149 |
| *CD86* | ACAAAAAGCCCACAGGAATG | TTAGGTTCTGGGTAACCGTGT | 160 |
| *MMP9* | CTGGGCAGATTCCAAACCT | TGTACACGCGAGTGAAGGTG | 172 |
| *CD19* | GCCTCCTCTTCTTCCTCCTC | AAGGGTTTAAGCGGGGACT | 169 |
| *CD20* | GCCTGGACTACACCACTCAC | AAAACTCCTGAGTCTCCAAGGC | 196 |
| *FLT3* | GGTTCCTGGTCAAGTGCTGT | GCAGGGTTAAAACGACAATGA | 156 |
| *RUNX2* | TCCTCCCCAAGTAGCTACCT | TACTGGGATGAGGAATGCGC | 152 |
| *NR3C1* | CTGGTGTGCTCTGATGAAGC | CCAGCCTGAAGACATTTTCGA | 191 |

**SUPPLEMENTARY TABLE S2**

Mouse DNA primers used for quantitative real-time PCR

| **Mouse gene** | **Forward 5’ – 3’** | **Reverse 5’ – 3’** | **Amplicon size (bp)** |
| --- | --- | --- | --- |
| *Rpl38* | AGGATGCCAAGTCTGTCAAGA | TCCTTGTCTGTGATAACCAGGG | 100 |
| *Ppia* | CAAATGCTGGACCAAACACAAACG | GTTCATGCCTTCTTTCACCTTCCC | 110 |
| *Hdac7* | GGAACACTTTCCCTTGCGTA | CTTCCTGAGCAGGGGATTCT | 103 |

**SUPPLEMENTARY TABLE S3**

Summary of high-throughput data used in this study and corresponding sources

| **Type of data** | **Samples** | **Treatment** | **Source** |
| --- | --- | --- | --- |
| RNA sequencing | t(4;11) pro-B-ALL SEM-K2 cells | DMSO | Data from this study (GSE268574) |
|  |  | MI-538 + chidamide |  |
| RNA sequencing | t(4;11) primary pro-B-ALL | **-** | PRJEB23605 (available in European Nucleotide Archive) |
|  | *KMT2A*-germline pro-B-ALL | **-** |  |
| ChIP sequencing  KMT2A immunoprecipitation | t(4;11) pro-B-ALL blasts | **-** | GSE202449 |
|  | CCRF cell line | **-** | GSE83671 |
|  | KOPN cell line | **-** |  |
|  | SEM-K2 cell line | **-** | GSE74812 |
|  | RS4;11 cell line | **-** | GSE38403 |
| ChIP sequencing  KMT2A N-terminal immunoprecipitation | SEM-K2  KMT2A::AFF1 overexpression | siRNA control | GSE202568 |
|  |  | KMT2A KD |  |
| DNA methylation arrays | CD19^+^ B lymphocytes | **-** | E-MTAB-8505  (available in Array Express) |
|  | Healthy B cell progenitors | **-** |  |
|  | *KMT2A*-germline pro-B-ALL | **-** |  |
|  | t(4;11) pro-B-ALL | **-** |  |
|  | t(9;11) pro-B-ALL | **-** |  |

**SUPPLEMENTARY FIGURE LEGENDS**

**SUPPLEMENTARY FIGURE S1 – HDAC7 reduces leukemogenic capacity of t(4;11) pro-B-ALL cells and improves survival *in vivo***

**(A)** Hierarchical clustering of germline *KMT2A* and t(4;11) pro-B-ALL patients, according to their transcriptomic profiles, from publicly available database (Supplementary Ref. 10 – Agraz-Doblas *et al*.). Germline *KMT2A* patients are labeled as ALL-KMgerm_xx (in black, n=10), while t(4;11) pro-B-ALL patients are labeled as ALL-KA1_xx (in green, n=26). **(B)** As in (A), but KMT2A::AFF1 pro-B-ALL patients are labeled as ALL-KA1_xx_low/high (n=26), according to *HDAC7* expression (13 per group, labeled in red for “low” and blue for “high”). **(C)** Protein levels of HDAC7 after overexposure of blot in **Fig. 1A**. β-actin was used as loading control. **(D)** Protein levels of HDAC7 in SEM-K2 TetOnTight-HDAC7 cells (and corresponding empty vector control cells), compared to REH cells. Where indicated, cells were treated with 1µg/mL of doxycycline for 72h. β-actin was used as loading control. **(E)** Average engraftment of SEM-K2 TetOnTight-HDAC7 cells and empty vector control cells in peripheral blood (PB) before doxycycline treatment (Day 0) (n=10 mice per group). **(F)** As in (C), but at Day 35 after treatment initiation (n=5 mice in empty vector group; n=8 mice in HDAC7 group). Statistical significance is indicated as: *p < 0.05; **p < 0.01; ***p <0.001; or n.s. non-significant.

**SUPPLEMENTARY FIGURE S2 – Chromatin remodeler EZH2 represses HDAC7 in t(4;11) pro-B-ALL cells**

**(A)** Heatmap representation of the methylation status of the CpG sites within human *HDAC7* gene and its corresponding promoter region in pro-B-ALL cells with germline *KMT2A* (n=20), and t(4;11) and t(9;11) translocations (n=37 and n=12, respectively). Data from healthy B cell progenitors (n=6) and CD19^+^ B lymphocytes (n=1) is also included. Red-colored lines on the left indicate the CpG sites with differential methylation in t(4;11) samples. Green-shadowed area highlights a region located in *HDAC7* promoter containing two differentially-methylated CpG sites. Data is publicly available in ArrayExpress (accession number: E-MTAB-8505). **(B)** ChIP-qPCR data for the three regions of *HDAC7* promoter after immunoprecipitation with H3K4me3 and H3K27me3 antibodies in SEM-K2, RS4;11 and REH cells, along with corresponding anti-rabbit IgG, as control (n=3-4 per region). **(C)** ChIP-qPCR data for the three regions of HDAC7 promoter shown in (B) and **Fig. 2B** for SEM-K2, RS4;11, ALL-PO and REH cells, after immunoprecipitation with H3K27ac antibody, and the corresponding IgG as control (n=3-5 experiments per cell line). **(D)** Total protein levels of H3K27me3, H3K9me3 and H3K27ac in REH, SEM-K2, RS4;11 and ALL-PO cell lines. Histone 3 total was used as loading control. **(E)** As in (D), but for H3K4me3 histone mark. **(F)** Hierarchical clustering of germline *KMT2A* and t(4;11) pro-B-ALL patients. As in **Supplementary Figs. S1A** and **S1B**, but splitting t(4;11) pro-B-ALL patients according to *EZH2* expression. Patients in the *EZH2*low group (n=13) are labeled in red, and patients in *EZH2*high group (n=13), in blue. **(G)** ChIP sequencing data for *EZH2* gene in SEM-K2 overexpressing KMT2A::AFF1. Black track shows binding peaks after immunoprecipitation against N-terminal region of KMT2A in control cells, while dark purple track shows peaks after *KMT2A::AFF1* knockdown (KD). Light purple track shows differential peak between KD cells and control cells (in log2 scale). Results obtained from publicly available data (GSM7548119). **(H)** Gene-set enrichment analysis of *KMT2A* germline (in red) and t(4;11) pro-B-ALL (in blue) patients, for a set of curated genes repressed (*top panel*) and activated (*bottom panel*) by EZH2. Curated gene sets were obtained from Nuytten *et al*. (Supplementary Ref. 7). **(I)** As in (H), but using a curated gene set Lu *et al*. (Supplementary Ref. 8). Results in (B) and (C) are shown as average ± SE. Statistical significance is indicated as: *p < 0.05; **p < 0.01; ***p <0.001; or n.s. non-significant.

**SUPPLEMENTARY FIGURE S3 – EZH2 blockade triggers HDAC7 expression in t(4;11) pro-B-ALL cells**

**(A)** Protein levels of EZH2 and total H3K27me3 in RS4;11 cells treated with increasing doses of GSK126 (*left panel*) and EPZ6438 (*right panel*), or DMSO solvent as control. In both cases, β-actin was used as loading control. **(B)** Protein levels of HDAC7 in RS4;11, SEM-K2 and ALL-PO cells treated with increasing doses of EPZ6438, or DMSO as solvent control. β-actin was used as loading control. **(C)** Expression of *PRDM1* and *CCND1* mRNA by qRT-PCR in SEM-K2 cells with increasing doses of GSK126, compared to DMSO as solvent control. *GAPDH* and *RPL38* were used as housekeeping genes (n=4 per condition). **(D)** As in (C), but using increasing doses of EPZ6438. Results in (C) and (D) are shown as average ± SE. Statistical significance is indicated as: *p < 0.05; **p < 0.01; ***p <0.001; or n.s. non-significant.

**SUPPLEMENTARY FIGURE S4 --- EZH2 inhibitors exert a weak effect on t(4;11) pro-B-ALL cells viability due to the induction of FLT3 and its downstream signaling**

**(A)** Cell viability by MTT assays in SEM-K2 (*left panels*) and RS4;11 (*right panels*) cells. Where indicated, cells were treated with increasing doses of GSK126 or EPZ6438 for 96 h. Results are presented as relative absorbance for the differences between values measured at 560 and 750 nm (n=6 per condition). **(B)** Expression of *FLT3* mRNA by qRT-PCR in SEM-K2 cells with increasing doses of GSK126 (*top panel*) or EPZ6438 (*bottom panel*) compared to DMSO as solvent control. *GAPDH* and *RPL38* were used as housekeeping genes (n=6-8 per condition). **(C)** Protein levels of phosphorylated STAT5 (pSTAT5) and total STAT5 in SEM-K2 cells after treatment with increasing doses of GSK126, or DMSO as solvent control, using β-actin as loading control (*top panel*). In *bottom panel*, quantification of pSTAT5/STAT5 ratio, setting DMSO condition as 1.00. **(D)** As in (C), but using increasing doses of EPZ6438. Results in (A) and (B) are shown as average ± SE. Statistical significance is indicated as: *p < 0.05; **p < 0.01; ***p <0.001; or n.s. non-significant.

**SUPPLEMENTARY FIGURE S5 – Inhibition of KMT2A::AFF1 cofactors reduces proliferation and leukemogenic capacity of t(4;11) pro-B-ALL cells**

**(A)** Inhibitory concentration 50 (IC50) curves for MI-538 compound obtained by MTT assay in SEM-K2, RS4;11 and REH cell lines. Chart is representative of 5 independent experiments. Values of the table and statistics were calculated based on the average of four independent experiments. **(B)** Expression of *HDAC2* and *HDAC3* mRNA by qRT-PCR in SEM-K2 cells treated with 1µM of chidamide for 48h, compared to DMSO as solvent control. *GAPDH* and *RPL38* were used as housekeeping genes (n=4 per condition). **(C)** Cell viability by MTT assays in RS4;11 cells. Where indicated, cells were treated with Menin inhibitors MI-463, MI-503 or MI-538 (1µM, 6 days) and/or chidamide (1µM, 48 h). Results are presented as relative absorbance for the differences between values measured at 560 and 750 nm (n=9 for MI-538 and n=3 for MI-463 and MI-503). **(D)** As in (C), but for REH cells (n=9 for MI-538 and n=6 for MI-463 and MI-503). **(E)** Representative macroscopic image of SEM-K2 colony formation assay plates in **Fig. 3C**. Results in (B-D) are shown as average ± SE. Statistical significance is indicated as: *p < 0.05; **p < 0.01; ***p <0.001; or n.s. non-significant.

**SUPPLEMENTARY FIGURE S6 – Combinatorial MI-538 + chidamide treatment impairs t(4;11) pro-B-ALL cells viability by reverting EZH2-mediated HDAC7 repression**

**(A)** Expression of *EZH2* mRNA by qRT-PCR in SEM-K2, RS4;11 and REH cells treated with 1µM of MI-538 for 6 days and/or 1µM of chidamide for 48h, compared to DMSO as solvent control. *GAPDH* and *RPL38* were used as housekeeping genes (n=4-6 per condition). **(B)** Protein levels of HDAC7 in SEM-K2 cells treated with Menin inhibitors alone or in combination with chidamide, or DMSO as solvent control. Where indicated, cells were treated with MI-463, MI-503 (1µM for 6 days) and/or chidamide (1µM for 48 h). β-actin was used as loading control. **(C)** Expression of *CD86* and *MMP9* genes mRNA by qRT-PCR in SEM-K2 treated with 1µM of MI-538 for 6 days alone or in combination with 1µM of chidamide for 48h, compared to DMSO as solvent control. *GAPDH* and *RPL38* were used as housekeeping genes (n=5-8 per condition). **(D)** Expression of *HDAC7* mRNA by qRT-PCR in SEM-K2-scrambled and SEM-K2-sh*HDAC7* cells. *GAPDH* and *RPL38* were used as housekeeping genes (n=4 per condition). **(E)** Protein levels of HDAC7 in SEM-K2-scrambled and SEM-K2-sh*HDAC7* cells. β-actin was used as loading control. **(F)** Cell viability by MTT assays in SEM-K2-scrambled and SEM-K2-sh*HDAC7* cells, treated for 6 days with 1µM of MI-538 and/or chidamide (1µM, 48 h), or DMSO solvent as control (n=9 per condition and cell line). Results in (A), (C), (D) and (F) are shown as average ± SE. Statistical significance is indicated as: *p < 0.05; **p < 0.01; ***p <0.001; or n.s. non-significant.

**SUPPLEMENTARY FIGURE S7 – MI-538 and chidamide confer a more differentiated B cell profile and increased dexamethasone sensitivity to t(4;11) pro-B-ALL cells**

**(A)** Gene-set enrichment analysis of SEM-K2 cells treated with MI-538 and chidamide (in red) or DMSO (in blue), for Menin inhibition gene signature obtained from He *et al*. (Supplementary Ref. 9). **(B)** Normalized counts from RNA-seq data in **Fig. 5A** for *HDAC7* and *EZH2* transcripts. Normal distribution of the data was verified using Shapiro-Wilk test. Student’s t-test was used to analyze significance. **(C)** As in **Fig. 5H**, mean fluorescence intensity for CD19 was analyzed in *KMT2A* germline PDX samples, after treatment with MI-538 + chidamide, or DMSO as control (n=4 per condition). **(D)** mRNA expression of *NR3C1* by qRT-PCR in SEM-K2 cells treated with 1µM of MI-538 for 6 days alone or in combination with 1µM of chidamide for 48h, compared to DMSO as solvent control. *GAPDH* and *RPL38* were used as housekeeping genes (n=5 per condition). Results in (C) and (D) are shown as average ± SE. Statistical significance is indicated as: *p < 0.05; **p < 0.01; or n.s. non-significant.

**SUPPLEMENTARY FIGURE S8 – MI-538 and chidamide reduce leukemogenic capacity of primary t(4;11) pro-B-ALL cells**

**(A)** Evolution of t(4;11) pro-B-ALL cells engraftment in peripheral blood (PB) from Day 0 (before treatment) to Day 42 (endpoint) for control (black dots), VxL (grey dots), VxL + MI-538 (blue dots), VxL + chidamide (yellow dots) and VxL + MI-538 + chidamide (red dots) treatment groups, plotted individually. Only PDX cells from t(4;11) pro-B-ALL #1 were used. **(B)** Measurement of spleen weight (compared to animal initial weight) for mice in all treatment groups at experimental endpoint. **(C)** As in **Fig. 7H**, but for murine *Hdac7* expression. *Rpl38* and *Ppia* were used as housekeeping genes (n=5-8 per group). Statistical significance is indicated as: *p < 0.05; **p < 0.01; ***p <0.001; or n.s. non-significant.
